# The Effect of Mindfulness-based Programs on Cognitive Function in Adults: A Systematic Review and Meta-analysis

**DOI:** 10.1007/s11065-021-09519-y

**Published:** 2021-08-04

**Authors:** Tim Whitfield, Thorsten Barnhofer, Rebecca Acabchuk, Avi Cohen, Michael Lee, Marco Schlosser, Eider M. Arenaza-Urquijo, Adriana Böttcher, Willoughby Britton, Nina Coll-Padros, Fabienne Collette, Gaël Chételat, Sophie Dautricourt, Harriet Demnitz-King, Travis Dumais, Olga Klimecki, Dix Meiberth, Inès Moulinet, Theresa Müller, Elizabeth Parsons, Lauren Sager, Lena Sannemann, Jodi Scharf, Ann-Katrin Schild, Edelweiss Touron, Miranka Wirth, Zuzana Walker, Ethan Moitra, Antoine Lutz, Sara W. Lazar, David Vago, Natalie L. Marchant

**Affiliations:** 1 Division of Psychiatry, University College London, London, UK; 2 School of Psychology, University of Surrey, Guildford, UK; 3 Department of Psychological Sciences, University of Connecticut, Storrs, CT USA; 4 Geneva School of Social Sciences, and Swiss Center for Affective Sciences, University of Geneva, Geneva, Switzerland; 5 Department of Radiology, Mayo Clinic, Rochester, MN USA; 6 German Center for Neurodegenerative Diseases (DZNE), Dresden, Germany; 7 Warren Alpert Medical School of Brown University, Providence, RI USA; 8 Alzheimers Disease and Other Cognitive Disorders Unit, Hospital Clínic, Institut d Investigacions Biomèdiques August Pi I Sunyer (IDIBAPS), Barcelona, Spain; 9 GIGA-CRC, In Vivo Imaging, Universite de Liege, Liege, Belgium; 10 INSERM UMR-S U1237, Caen-Normandie University, GIP Cyceron, Caen, France; 11 School of Public Health, Brown University, Providence, RI USA; 12 Department of Psychiatry, Medical Faculty, University of Cologne, Cologne, Germany; 13 Essex Partnership University NHS Foundation Trust, Wickford, UK; 14 Lyon Neuroscience Research Center, INSERM U1028, CNRS UMR5292, Lyon 1 University, Lyon, France; 15 Athinoula A. Martinos Center for Biomedical Imaging, Massachusetts General Hospital, Boston, MA USA; 16 Department of Physical Medicine & Rehabilitation, Vanderbilt University Medical Center, Nashville, TN USA

**Keywords:** Mindfulness, Meditation, Intervention, Neuropsychology, Elder, Aging

## Abstract

Mindfulness-based programs (MBPs) are increasingly utilized to improve mental health. Interest in the putative effects of MBPs on cognitive function is also growing. This is the first meta-analysis of objective cognitive outcomes across multiple domains from randomized MBP studies of adults. Seven databases were systematically searched to January 2020. Fifty-six unique studies (*n* = 2,931) were included, of which 45 (*n* = 2,238) were synthesized using robust variance estimation meta-analysis. Meta-regression and subgroup analyses evaluated moderators. Pooling data across cognitive domains, the summary effect size for all studies favored MBPs over comparators and was small in magnitude (*g* = 0.15; [0.05, 0.24]). Across subgroup analyses of individual cognitive domains/subdomains, MBPs outperformed comparators for executive function (*g* = 0.15; [0.02, 0.27]) and working memory outcomes (*g* = 0.23; [0.11, 0.36]) only. Subgroup analyses identified significant effects for studies of non-clinical samples, as well as for adults aged over 60. Across all studies, MBPs outperformed inactive, but not active comparators. Limitations include the primarily unclear within-study risk of bias (only a minority of studies were considered low risk), and that statistical constraints rendered some *p*-values unreliable. Together, results partially corroborate the hypothesized link between mindfulness practices and cognitive performance. This review was registered with PROSPERO [CRD42018100904].

**Supplementary Information:**

The online version contains supplementary material available at 10.1007/s11065-021-09519-y.

## Introduction

Mindfulness-based programs (MBPs) primarily target improved mental health and wellbeing (de Vibe et al., 2017). Notably, MBPs not only support the development of emotional, but also attentional self-regulation (Lutz et al., 2008). Indeed, mindfulness can be broadly defined as ‘paying attention to the present moment’ (Kabat-Zinn, 2003). The importance of attention in mindfulness practice and theory has been formally described by neurocognitive frameworks. In order to delineate relevant mechanisms, frameworks deconvolve mindfulness into component parts. In addition to attention regulation, these include intention and motivation, emotion regulation, extinction and reconsolidation of maladaptive behavior, changes in perspectives on self, and interoception (Shapiro et al., 2006). The theorized relationship of these components to specific neurocognitive systems has been outlined in detail (Hölzel et al., 2011; Lutz et al., 2015; Vago & Silbersweig, 2012). The implication of this is that development of greater mindfulness capacity may manifest in parallel with improved cognitive function.

The primary element included in MBPs to support the development of greater mindfulness capacity is the teaching of formal practices, namely: the body scan, mindful movement and sitting meditation (Crane et al., 2017). Whilst each of these practices may contribute to improved cognitive function, the vast majority of theory focuses exclusively on sitting meditation. The types of sitting meditation typically included in MBPs are predominantly focused attention and open monitoring practices. Focused attention meditation involves focusing on an ‘object’ (e.g. breathing-related sensations), whilst open monitoring meditation is characterized by the receptive monitoring of experience. Both types of meditation require practitioners to disengage from distractors (e.g. mind wandering) which interfere with the intended foci, and can thus be considered practices of attentional self-regulation (Lutz et al., 2008). Based on this understanding, a considerable number of studies have evaluated if and how participation in MBPs translates to improved performance on objective measures of cognitive function.

A seminal systematic review in this field (Chiesa et al., 2011) concluded that the preliminary evidence suggested mindfulness training improves attention. In contrast, a review of mindfulness-based stress reduction (MBSR) and mindfulness-based cognitive therapy (MBCT) – two of the most widely employed MBPs – found no effect on attention but did report improvements in working memory (Lao et al., 2016). Most recently, Cásedas et al. (2020) conducted a meta-analysis focusing exclusively on executive function outcomes from randomized mindfulness meditation studies, concluding that mindfulness meditation outperformed comparators (*g* = 0.34). Subdomains of executive function were also examined; MBPs outperformed comparators on working memory (*g* = 0.42) and inhibitory control outcomes (*g* = 0.42), but not for cognitive flexibility. While Cásedas et al. (2020) undertook the first quantitative synthesis in the field, important questions remain. Cognitive domains beyond executive function were not evaluated, and there was considerable variability in the age and clinical status of study samples. Meditation interventions were also highly variable – the briefest intervention included three 20-min classes, whilst the longest comprised a three-month meditation retreat. The potential impact of this variability on the estimated effects is unknown, as the relatively small number of meta-analyzed studies (*k* = 13) precluded the evaluation of putative moderators via meta-regression or subgroup analyses.

The current review addressed the limitations of Cásedas et al. (2020) through the inclusion of multiple cognitive domains, focusing on a more homogeneous set of interventions, and evaluating a range of potential moderators, including age and clinical status. Age is a risk factor for both subclinical and clinically relevant cognitive decline (Anderson & Craik, 2017; van der Flier & Scheltens, 2005); hence older adults’ cognitive function is typically somewhat worse relative to earlier adulthood. It is plausible that age could moderate the effect of MBPs on cognition, given that there could be less of a ‘ceiling effect’ to limit potential improvements in older adults. Participants’ clinical status (i.e. whether they have a diagnosis or not) may also moderate MBP effects on cognition, perhaps via a similar association with initial cognitive performance, but perhaps also due to other aspects of living with illness, which might render full participation in an MBP more difficult.

### Aims

The primary aim of the current review was to provide a comprehensive overview and meta-analysis of the effect of MBPs on cognitive performance in adults using results from randomized controlled studies. Additional aims were to estimate effects for separate cognitive domains and subdomains, for adults (< 60 years) and older adults (≥ 60 years) separately, and for clinical and non-clinical study samples separately.

## Method

### Protocol and Registration

In line with the Preferred Reporting Items for Systematic Reviews and Meta-Analyses (PRISMA) recommendations (Moher et al., 2009), this review was registered with PROSPERO in July 2018 [CRD42018100904].

### Eligibility Criteria

To be eligible, studies had to be randomized, written in English, and could be published or unpublished (the latter comprising dissertations and theses). Study comparators could be active or inactive. Study samples had to comprise adults with a minimum mean age of 18 years; both clinical and non-clinical samples were eligible. Studies had to include an MBP, defined as an intervention that was mindfulness-based; comprised of four or more sessions; and delivered in-person, by a facilitator, to groups of participants. This definition drew heavily on a framework outlining MBP characteristics (Crane et al., 2017), which implicitly pertains to MBPs with eight or more sessions. For this review, studies were required to have a minimum number of four sessions based on previous research that adjudged four sessions to be an adequate minimal dose (Williams et al., 2014) and evidence that as few as four mindfulness sessions can improve cognitive performance (Zeidan et al., 2010). Permitting greater variability in MBP session number also facilitated the evaluation of this variable as a moderator. Considering other MBP characteristics, the in-person, group-based nature of MBPs is thought to support participants’ learning. MBPs exclusively delivered remotely or digitally were therefore ineligible. Studies where the setting was exclusively residential (i.e. a mindfulness retreat) were excluded in order to reduce variability between interventions. Although retreats and MBPs both involve mindfulness practice and teaching, the residential nature of the former engenders a more intensive experience. Lastly, the following interventions were not considered MBPs for the purpose of this review: integrative body-mind training, acceptance and commitment therapy, dialectical behavior therapy, compassion-based interventions, loving-kindness meditation, mantra meditation, yoga, qi gong or tai chi. While we consider these interventions to be mindfulness-informed, they are not mindfulness-based in the sense of the above definition.

We exclusively examined the effect of MBP participation on objective measures of cognitive performance. Here, objective is defined as behaviorally-measured performance on neuropsychological or laboratory-based cognitive tests and includes both pen-and-paper and computerized paradigms. Self-reported measures were thus excluded. To be included, studies must have administered one or more measures of an eligible cognitive domain pre- and post-intervention. Eligible cognitive domains were attention, perception, declarative memory, language, construction, reasoning, and executive function. Tests of cognitive function used to screen for mild cognitive impairment (MCI) and dementia, for example the mini-mental state examination (MMSE; Folstein et al. (1975)) were also included; this category was denoted the ‘cognitive aging’ domain. Whilst these measures can be used to screen for early-onset cognitive decline (i.e. in midlife), in this review they were exclusively administered to older adults (mean age ≥ 60 years).

We excluded cognitive tests that included affective components (e.g. emotional variants of the Stroop test). This was justified on the basis that these were less common than non-affective cognitive tests, could suffer from the confounding effect of emotional valence, and exhibited significant variability in content and administration. We also excluded studies which measured cognition immediately following mindfulness practice (the majority, in any case, reporting the effects of a single mindfulness session). The rationale for this was that measuring cognition immediately post-practice could be conceptualized as capturing transient ‘state’ mindfulness effects, whereas the current focus was on potentially more stable effects.

### Search Strategy

The electronic databases AMED, CINAHL Plus, Embase, Medline, PsycBOOKS, PsycINFO, Scopus and Web of Science were systematically searched. Additionally, grey literature searches were conducted with ProQuest Dissertations and Theses Global, ClinicalTrials.gov and Google Scholar (Haddaway et al., 2015). Lastly, references from other reviews were hand-screened by two experts from the mindfulness research field (DV, SWL).

Briefly, search strings combined the stem ‘mindful*’ with interventional terms adapted from other reviews (Bhome et al., 2018; Smart et al., 2017; Verbeek et al., 2005), and cognitive domain terms derived from a handbook of neuropsychological assessment (Lezak, 2012). The initial search was conducted on December 2nd, 2018 and updated on January 23rd, 2020. Please see the supplementary materials for database-specific search strings.

### Study Selection and Data Extraction

The web platform Covidence (Veritas Health Innovation (Melbourne) was used for deduplication, and to coordinate multiuser title-abstract and full-text screening. Each study record was screened in duplicate by two reviewers independently at both stages. Disagreements were resolved by a third reviewer. Pairs of reviewers independently extracted study data into a piloted form in duplicate; these two versions of the data were then compared. Where discrepancies arose, TW checked the relevant publication, and confirmed one of the previously extracted values. In rare cases where TW did not agree with either of the discrepant values, the final value was settled by at least one other member of the review team (in the first instance, EM).

### Coding Scheme

For descriptive and analytic purposes, studies were coded as ‘adult’ (mean sample age < 60 years) or ‘older adult’ (mean sample age ≥ 60 years). The rationale for selecting this age cut-off was that age-related cognitive decline typically manifests during the seventh decade of life (Cornelis et al., 2019; Schaie et al., 2004). Dichotomizing age therefore enabled us to explore whether MBPs might improve or restore cognitive abilities. MBP types were coded into three categories. The first category, ‘unmodified MBSR/MBCT’, represented versions of those interventions delivered according to the original protocols. Unmodified MBSR/MBCT were grouped together as they share a similar structure, and are arguably the most influential and established MBPs (Crane et al., 2017). The second category, ‘modified MBPs’, coded for interventions described by study authors as being variously adapted from MBSR/MBCT. The third category, ‘generic MBPs’, coded for MBPs described without any reference to MBSR/MBCT. Crane et al. (2017) note that MBPs typically incorporate three formal mindfulness practices: the body scan, mindful movement and sitting meditation, as well as a retreat day (the latter is sometimes omitted from research studies due to resource constraints). We thus recorded the number of formal mindfulness practices and retreats included in each MBP. Studies solely utilizing waitlist, treatment as usual, or ‘no intervention’ control groups were coded as ‘inactively-controlled’; all other studies were coded as ‘actively-controlled’. We did not specify an a priori coding scheme for subclassifying active comparator interventions, instead operationalizing this post-hoc.

Two reviewers independently coded outcomes into cognitive domains during data extraction; this was informed by professional experience, test documentation and the wider academic literature. It was common for a given test to yield multiple outcomes – reviewers could code these outcomes into separate domains as appropriate. Three domains were further divided into subdomains. For executive function, these were cognitive flexibility, working memory (for measures requiring both informational maintenance and manipulation), and inhibition, after Diamond (2013). For attention, these were alerting and orienting, after Petersen and Posner (2012); the third attentional subdomain specified by that framework – executive control – was here merged with the inhibition subdomain of executive function given the significant overlap between these constructs. The declarative memory domain was divided into the episodic memory subdomain (combining immediate and delayed recall outcomes) and the short-term memory subdomain (for measures requiring the maintenance, but not manipulation, of information).

A number of the included tests of executive function and attention are scored according to participants’ reaction time or accuracy. Typical examples include the Attention Network Test (Fan et al., 2002) and the Continuous Performance Test (Cohen, 2011). We encountered significant variability between studies in the types of scores reported for these tests, even for the same or similar measures. Namely, authors reported scores across the following categories: (i) individual types of trial (e.g. incongruent trials) or types of response (e.g. correct hits); (ii) performance collapsed across conditions (e.g. global mean reaction time); or (iii) summary scores (contrasts between different types of trial/response, or other summary indices). Similarly, some studies reported reaction time but not accuracy scores (or the converse) or appeared to report scores incompletely (e.g. reporting data for correct hits but not false alarms). For these types of executive function and attention measures (i.e. those scored using reaction time/accuracy), only summary scores (i.e. (iii)) were meta-analyzed. This was justified on the basis that summary scores are a function of individual scores, rendering analyses including both invalid; the majority of studies included summary scores; and, particularly for executive function, contrasts are considered crucial to isolate the cognitive processes of interest. A good example of an executive function measure that utilizes contrast scoring is the Stroop test. The present approach thus maintained the independence of (while maximally exploiting) outcome data; reduced variability amongst score types; and facilitated the interpretation of results.

### Risk of Bias in Individual Studies

Risk of bias at the level of individual studies was assessed during data extraction using the Cochrane risk of bias tool (Higgins et al., 2011), yielding pairs of independent ratings. The Cochrane tool assesses risk of selection bias, performance bias, detection bias, attrition bias, reporting bias, and other potential biases. For each of these domains, reviewers judged the risk of bias as ‘Low’ (bias unlikely to alter the results seriously), ‘Unclear’ (indicating lack of information, or uncertainty over potential bias) or ‘High’ (bias with the potential to alter the results seriously). Disagreements were resolved using the same approach taken for data extraction.

## Statistical Analyses

### Calculation of Effect Sizes

The measure of effect size was the standardized mean difference, with a correction factor applied for small sample sizes (Morris, 2007). For cognitive tests where lower scores indicate better performance, we multiplied scores by minus one, so that all scores followed the format of higher values reflecting better performance. For each cognitive outcome score, we calculated: (i) the pre- to post-intervention (i.e. immediately following the conclusion of the intervention) mean difference for each study arm, and then (ii) the difference between these change scores. This score was then divided by the pooled pre-test standard deviation, and adjusted using approximate correction factor *J,* to give Hedges’ *g* (Morris, 2007). The calculation of *g* (see supplementary materials for the presently used formula) requires knowledge of the pre- to post-intervention correlation for each outcome. These correlations were only available for four studies, with these having a mean of *r* = 0.49. Given this empirical observation, and that a value of *r* = 0.50 is typically substituted for unknown correlations, we used the latter value for all effect size calculations. Some studies included both an inactive and an active comparator, in addition to the MBP. For studies with both types of comparator, we only included effect sizes versus active comparator interventions in the main analyses, given this constitutes a more rigorous evaluation of MBPs. However, for subgroup analyses reporting comparisons for MBPs versus active and inactive comparators separately, we included comparisons between the MBP and *all* available comparators. Consequently, the total number of studies and effect sizes across these subgroups exceeded the total numbers included in the main analyses.

### Accounting for Dependencies

Many studies reporting the effects of MBP participation on cognition administered more than one outcome measure meeting the eligibility criteria. Conventional meta-analysis cannot optimally accommodate this type of data, as an assumption of this approach is that each outcome is sampled independently. Conventional solutions to this problem include selecting a single effect size, or the mean effect size, for each study. Both approaches lose information, and are therefore not recommended (Matt & Cook, 1994).

Random-effects meta-analysis with robust variance estimation was therefore used for quantitative syntheses. This method can model multiple effect sizes from related measures that are clustered within studies without averaging data, and also controls for the dependence between these effects (Hedges et al., 2010). The robust variance estimation meta-analysis was conducted with the ‘robumeta’ package in R version 3.6.0, with small-sample corrections enabled (Fisher & Tipton, 2017). As per the default option for the robumeta package, rho (presumed correlation amongst different outcomes within studies) was set to 0.8, and sensitivity analyses varied rho from 0–1 to determine the effect these values could have on *Tau*^*2*^. Significant results were defined as those having a *p*-value < 0.05. Notably, *p*-values for robust variance estimation meta-analytic estimates are unreliable where the model degrees of freedom < 4 (Fisher & Tipton, 2017). We highlight where this is the case and do not report these *p*-values. For the primary meta-analysis, data from all studies and all cognitive domains were pooled.

### Heterogeneity

Heterogeneity for each model is reported using *Tau*^*2*^, which represents between-study variance, and *I*^*2*^, which represents the proportion of observed dispersion due to real variation in effect sizes, rather than random error.

### Moderator and Subgroup Analyses

A series of meta-regressions evaluated whether the following variables (possible values) moderated effect sizes:

(i) Type of comparator (active; inactive)
(ii) Age group (adults, mean age < 60 years; older adults, mean age ≥ 60 years)
(iii) Clinical status (clinical; non-clinical)
(iv) Type of MBP (unmodified MBSR/MBCT; modified MBP; generic MBP)
(v) Number of formal mindfulness practices (i.e. how many of the following were included: body scan, mindful movement, and sitting meditation)
(vi) Retreat included (yes; no)
(vii) Number of MBP sessions (continuous variable)
(viii) Frequency of MBP sessions (number per week, continuous variable)
(ix) Duration of MBP sessions (minutes, continuous variable)

For meta-regression, we only used the main comparator (see ‘Calculation of effect sizes’). Each moderator was analyzed in a univariable meta-regression, and then the nine variables were simultaneously entered in a multivariable meta-regression. Subgroup analyses were also used to estimate pooled effect sizes for different categories within each of the following variables (coded as per meta-regression): age group; clinical status; MBP type; and type of comparator. We also conducted subgroup analyses of separate cognitive domains and subdomains. The broad range of moderators and domains evaluated resulted in multiple statistical comparisons, which can inflate the Type I error rate. One mitigatory approach is to use a Bonferroni-type correction to the statistical significance threshold. However, there is a lack of consensus regarding the suitability of this method for meta-analysis. Given this, we utilized an alternative approach, making a clear distinction between planned and exploratory subgroup analyses, as suggested by Pigott and Polanin (2020).

### Testing for and Managing Publication Bias

We employed well-established methods to assess for the presence of small study effects. Whilst publication bias is one explanation for small study effects, other causes exist. For example, it has been suggested that larger, more expensive trials are more likely to be methodologically rigorous, which may result in smaller effect sizes (Sterne et al., 2000). We assessed for the presence of small study effects using a regression-based method, meta-regressing effect size standard error on effect size. A significant association between effect sizes and their standard errors constitutes evidence of small study effects. The intercept from this model can be interpreted as the predicted value of an effect size with a standard error of zero (i.e. that which would be obtained for a hypothetical, infinitely large study). The intercept can thus be considered a measure of effect size adjusted for small study effects (Moreno et al., 2009).

## Results

### Study Selection

**Fig. 1:**
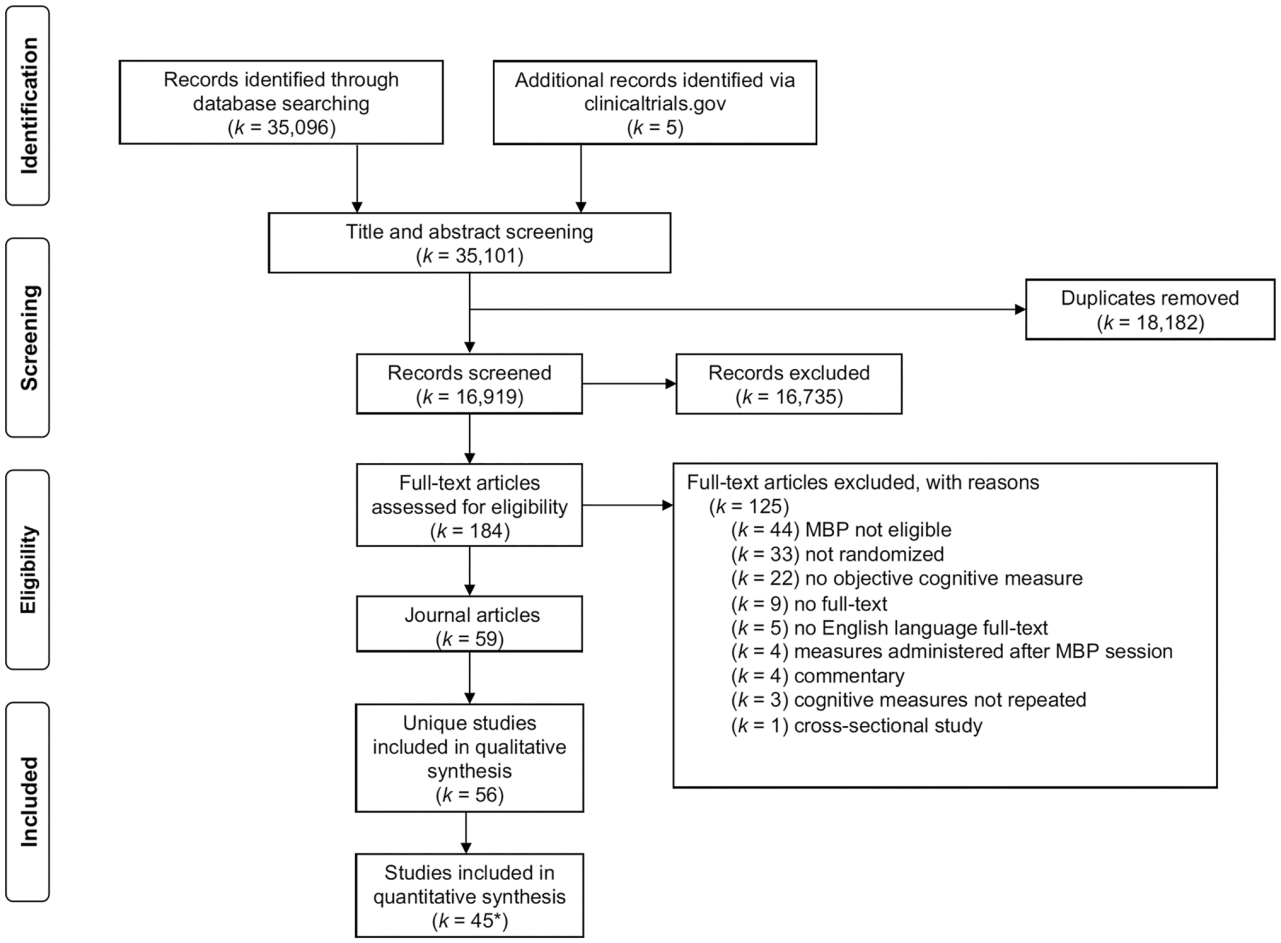
PRISMA flowchart. *k* Number of studies, *MBP* Mindfulness-based program. *Ten studies did not present data in a format amendable to meta-analysis, and a further study (Rothschild et al., 2017) was excluded from the final meta-analysis

The literature search across seven databases yielded a total of 35,101 title-abstracts through January 2020. After deduplication, 16,919 title-abstracts remained. Each title-abstract was screened by TW and one other reviewer (EP, ML, AC or MS), with 184 title-abstracts subsequently included for full-text review. Each full-text was screened by TW and one other reviewer (ML, AC or MS). During the full-text retrieval and data extraction process, another 125 titles were excluded (see Fig. 1 for PRISMA flowchart), with a final total of 59 articles being included.

### Study Characteristics

Characteristics of individual studies are presented in Table 1 and summarized in supplementary Table S1. In three cases, different results from the same study were reported across two articles. In these cases, both sets of results were merged into a single study record. This resulted in the inclusion of 56 unique study samples in the final review, representing a total of 2,931 participants (1,489 in the MBP arms and 1,442 in the main comparator arms). Study sample sizes varied considerably from 14 to 200 participants (median 43).

**Table 1:** Randomized controlled studies of mindfulness-based programs reporting cognitive outcomes

**Study**	**Sample size (*n*)**	**Mean age (years)**	**Participants**	**MBP name and type**	**Number of sessions**	**Session duration (mins)**	**Comparator type**^**#**^	**Assessed cognitive domains**^**#**^					
								**Executive function**	**Attention**	**Declarative memory**	**Cognitive aging**	**Visual perception**	**Construction**
Allen et al. (2012)	61	26.5	Non-clinical	MT^3^	6	120	ACI	♦	♦				
*Anderson et al. (2007)	72	39.2	Non-clinical	MBSR^1^	8	120	WL	♦	♦				
*Bachmann et al. (2018)	40	40.1	ADHD	MAP^2^	8	150	ACI			♦			
*Baird et al. (2014)	44	20.5	Non-clinical	MC^3^	8	45	ACI			♦		♦	
*Becerra et al. (2017)	46	33.9	Non-clinical	MP^3^	4	*NR*	WL	♦	♦				
*Biermann (2011)	42	73.4	Non-clinical	MM^3^	8	45–60	WL	♦					
*Bowden et al. (2012)	30	*NR*	Non-clinical	MT^3^	10	75	ACI	♦					
*Bubb (2014)	32	82.4	Non-clinical	mMBSR^2^	8	120	WL	♦	♦	♦	♦		♦
*Choles (2018)	58	41.2	Non-clinical	MBEB^2^	10	168	WL	♦					
*Churcher Clarke et al. (2017)	31	80.6	Dementia	MProg^2^	10	60	TAU				♦		
*Fam et al. (2020)	36	71.7	MCI	MAProg^2^	12	40	ACI			♦	♦		♦
*Flook et al. (2013)	18	43.1	Non-clinical	mMBSR^2^	8	150	WL		♦				
Giannandrea et al. (2019)	37	36.2	Non-clinical	MBSR^1^	9	*NR*	WL	♦	♦				
Greenberg et al. (2012); (Greenberg et al., 2013)	64	25.8	Non-clinical	mMBCT^2^	7	120	WL	♦					
Grossman et al. (2010)	150	47.3	MS	MBI^2^	8	150	NI	♦	♦	♦			
*Heredia et al. (2017)	25	21.2	Non-clinical	rMBTP^3^	4	120	WL	♦	♦	♦			
*(Isbel et al., 2019a, 2019b)	67	71.2	Non-clinical	MTech^2^	8	120	ACI		♦				
*Ives-Deliperi et al. (2013)	23	35.4	BD	MBCT^1^	8	*NR*	WL	♦		♦			♦
*Jensen et al. (2012)	32	25.0	Non-clinical	MBSR^1^	8	150	ACI	♦	♦	♦		♦	
*Jermann et al. (2013)	36	46.8	rMDD	MBCT^1^	8	120	NI	♦					
*Johansson et al. (2012)	26	55.5	Stroke/TBI	MBSR^1^	8	150	WL	♦	♦				
*Johns et al. (2016)	71	56.6	Cancer survivors	MBSR^1^	8	120	ACI	♦					
*Josefsson et al. (2014)	86	49.6	Non-clinical	MM^3^	7	45	ACI	♦					
*Klainin-Yobas et al. (2019)^€^	55	71.3	MCI	MAProg^3^	13	40	ACI				♦		
*Korponay et al. (2019)	70	49.1	Non-clinical	MBSR^1^	8	150	ACI	♦					
*(Kurmi et al., 2019a, 2019b)	100	63.9	Non-clinical	MM^3^	38	30	NI	♦	♦	♦			
*Larouche et al. (2018)	41	71.6	MCI	MBI^2^	8	150	ACI	♦	♦	♦			
*Lebares et al. (2019)	21	28.3	Non-clinical (medical)	mMBSR^2^	8	120	ACI	♦					
*Li et al. (2018)	30	29.4	Non-clinical	mMBCT^2^	8	150	ACI	♦					
*Lymeus et al. (2016)	35	25.5	Non-clinical	mMBSR^2^	8	90	WL		♦				
*Ma (2018)	41	38.8	Mild anxiety	mMBCT^2^	8	90	ACI		♦				
*MacCoon et al. (2014)	57	45.9	Non-clinical	MBSR^1^	8	158	ACI		♦				
*Manglani et al. (2020)	40	45.7	MS	MT^2^	4	120	ACI	♦	♦	♦			
*Martins (2012)	24	72.0	Non-clinical	MBSR^1^	8	120	WL	♦	♦				
*Mitchell et al. (2017)	20	38.6	ADHD	MAP^2^	8	150	WL	♦	♦	♦			
*Moynihan et al. (2013)	200	73.5	Non-clinical	MBSR^1^	8	120	WL	♦					
*Mrazek et al. (2013)	48	20.8	Non-clinical	MT^3^	8	45	ACI	♦					
*Oken et al. (2010)	21	64.9	Non-clinical (caregiver)	mMBCT^2^	6	90	ACI	♦	♦	♦			
*Payne (2017)	20	77.3	Dementia	mMBCT^2^	8	90	NI				♦		
Quan et al. (2018)	48	19.2	Non-clinical	mMBCT^2^	7	100	ACI	♦	♦				
*Roeser et al. (2013)	58	44.6	Non-clinical	MT^2^	9	150	WL	♦					
Rothschild et al. (2017)	123	19.1	Non-clinical (military)	MM^3^	72	30	NI		♦				
Schoenberg et al. (2014)	44	37.0	ADHD	mMBCT^2^	12	180	WL	♦	♦				
*Schöne et al. (2018)	34	21.1	Non-clinical	MBAM^3^	6	90	ACI		♦				
Smart et al. (2016)	38	69.8	SCD	mMBSR^2^	8	120	ACI	♦	♦				
Soler et al. (2016)	44	32.4	PD	MT^3^	10	150	ACI	♦	♦				
*Tang et al. (2015)	60	35.1	Epilepsy	MBT^2^	4	150	ACI	♦		♦			♦
*van den Hurk et al. (2012)	71	49.5	MDD	MBCT^1^	8	150	NI	♦	♦				
*Webb et al. (2018)^£^	37	19.9	HIV	mMBSR^2^	8	120	ACI	♦					
*Wells et al. (2013)	14	73.7	MCI	MBSR^1^	8	120	WL				♦		
*Wetherell et al. (2017)	103	72.0	Stress & cognitive disorders	mMBSR^2^	8	90	ACI			♦			♦
*Whitmoyer et al. (2020)	74	66.4	Non-clinical	MBAT^2^	4	90	ACI	♦	♦				
*Zanesco et al. (2019)	80	34.0	Non-clinical (military)	MBAT^2^	4	120	NI		♦				
*Zhang (2013)	69	25.3	Non-clinical (pregnant)	MMoth^2^	8	90	NI		♦				
Zhang et al. (2019)	36	22.5	Non-clinical	MT^2^	8	120	WL	♦					
Zhu et al. (2019)	48	24.2	Non-clinical	MBTra^2^	11	90–120	NI	♦	♦				

Sample size (*n*) only reflects the total participants across the MBP and main comparator arms

1-Main – *MBP* Mindfulness-based program, *NR* Not reported. 2-Participants – *ADHD* Attention Deficit Hyperactivity Disorder, *MS* Multiple Sclerosis, *BD* Bipolar Disorder, *MDD* Major Depressive Disorder, *rMDD* Remitted Major Depressive Disorder, *MCI* Mild Cognitive Impairment, *TBI* Traumatic Brain Injury, *SCD* Subjective Cognitive Decline, *PD* Personality Disorder, *HIV* Human Immunodeficiency Virus. 3-Intervention names – *MT* Mindfulness Training, *MAP* Mindful Awareness Practices, *MBSR* Mindfulness-Based Stress Reduction, *MAProg* Mindfulness Awareness Program, *MC* Meditation Class, *MP* Mindfulness Practice, *MProg* Mindfulness Program, *MM* Mindfulness Meditation, *mMBSR* Modified Mindfulness-Based Stress Reduction, *MBEB* Mindfulness Based Emotional Balance, *MBCT* Mindfulness-Based Cognitive Therapy, *mMBCT* Modified Mindfulness-Based Cognitive Therapy, *MBI* Mindfulness-Based Intervention, *rMBTP* Reduced Mindfulness-Based Training Program, *MTech* Mindfulness Technique, *MBAT* Mindful Breath Awareness Training, *MBATP* Mindfulness-Based Attention Training Program, *MBAM* Mindful Breath Awareness Meditation, *MBT* Mindfulness-Based Therapy, *MBTra* Mindfulness-Based Training, *MMoth* Mindful Motherhood. 4-Comparator types – *ACI* Active comparator intervention, *WL* Waitlist, *TAU* Treatment as usual, *NI* No intervention

^1^Unmodified MBSR/MBCT; ^2^Modified MBP; ^3^Generic MBP; *Included in the final meta-analysis; ^#^For the meta-analyzed studies, we only list the domains contributing data to quantitative syntheses; ^£^For this study, data were available for the subsample of participants with individual age ≥ 18 years (some participants were younger), these data alone were included in the review; ^€^For this study, outcomes were reported (i) following three months of weekly sessions, and (ii) after a further six months of monthly sessions. In order to maintain comparability with other MBPs, we only included data from the first timepoint; ^#^Here we list the main comparator included in analyses, additional inactive comparators were included in some subgroup analyses

Publication year ranged from 2007–2020. Half of the included studies (*k* = 28) were published in 2017 or later, highlighting the contemporary interest in the putative cognitive effects of MBPs. Twenty-four studies (43%) took place in North America, with the remaining studies taking place in Europe (*k* = 18; 32%), Asia (*k* = 9; 16%), Australia (*k* = 2; 4%), Israel (*k* = 2; 4%), and South Africa (*k* = 1; 2%). The majority of studies (*k* = 54; 96%) randomized participants at the individual level, while two studies (Rothschild et al., 2017; Zanesco et al., 2019) utilized cluster-randomization.

Forty-four studies (78%) did not follow up participants beyond the end of the intervention, while seven studies (13%) used short-term (8–18 week) follow-ups, and five studies (9%) used long-term (24–44 week) follow-ups (see supplementary materials for a narrative overview of follow-up results).

### Participant Characteristics

The mean age of included participants ranged from 19 to 82 years (median 41 years). Forty studies (71%) were of adults (< 60 years), while sixteen studies (29%) recruited older adults (≥ 60 years). The overall proportion of female participants was 67%. Twenty-two studies (39%) reported sample ethnicity data. Across these studies, 71% of participants were white, 15% were Asian, and 9% were black (the ethnicity of the remaining 5% was not reported or coded as ‘Other’). Thirty-four studies (61%) provided some data on the educational attainment of participants. Considering only the 16 (29%) studies which reported education in years, participants’ mean education ranged from four to 17 years (median 15 years). We pooled available age, sex, education, and ethnicity data for the MBP and main comparator arms separately (see supplementary Table S2). Inspecting the relevant means/proportions between arms broadly confirmed the effectiveness of randomization (i.e. groups were highly comparable across these characteristics).

Thirty-four studies (61%) recruited participants from non-clinical populations. Twenty-six (46%) of these recruited individuals from university or general community populations, whilst the remaining study samples represented specific professional groups (*k* = 6; 11%), caregivers of people with dementia (*k* = 1; 2%) or women during pregnancy (*k* = 1; 2%). Twenty-two studies (39%) recruited participants from clinical populations. These were broadly categorizable as comprising individuals with neurocognitive disorders (i.e. subjective or objective cognitive dysfunction, including dementia; *k* = 8; 14%), psychiatric disorders (*k* = 8; 14%), or neurological disorders (*k* = 4; 7%). A single study recruited persons who had recovered from cancer, while another included HIV-positive individuals. The preponderance of neurocognitive, psychiatric and neurological studies in this review is unsurprising, given that cognitive dysfunction is, by definition, present in neurocognitive disorders, and frequently implicated in psychiatric and neurological disorders.

Thirty-six (64%) studies reported information about participants’ prior or current experience with mindfulness practices. Twenty-six studies (46%) addressed this under study eligibility criteria, stipulating that previous meditation experience (*k* = 17; 30%), or current meditation practice (*k* = 9; 16%), were exclusionary. Nine studies (16%) took a descriptive approach, with six studies (11%) stating that all participants were meditation naïve, and three studies (5%) stating that some participants had prior experience of meditation.

### Intervention Characteristics

Ten studies (18%) used unmodified MBSR, while three (5%) used unmodified MBCT. Thirty studies (54%) featured modified MBPs. Typical modifications included reducing the number of sessions or omitting the retreat day; reducing the duration of sessions to facilitate the participation of attentionally-impaired individuals; adapting the psychoeducational content for non-stressed/non-depressed samples; and the omission of the mindful movement practice for persons with reduced mobility. The remaining interventions (*k* = 13; 23%) were coded as generic MBPs. In general, generic MBPs lacked the psychoeducational components common to the other MBP types; these interventions thus predominantly featured sessions of a shorter duration, primarily focusing on mindfulness practice.

The number of sessions included by MBPs ranged from four to 72, with most programs (*k* = 47; 84%) being delivered over six to 12 sessions. The majority of interventions (*k* = 42; 75%) were between six and 12 weeks in length, highlighting the convention of delivering MBP sessions weekly. Fifty-three studies (95%) reported the duration of in-person, group-based MBP sessions, which ranged from 30 to 180 min, with the majority (*k* = 40; 71%) having durations between 90 and 150 min. The MBP included a retreat day in 16 studies (29%) and did not include a retreat in 11 studies (20%). The remaining studies (*k* = 29; 51%) were unclear regarding MBP retreat provision. Including both sessions and retreats, total MBP intervention duration ranged from 315 to 2,190 min (median 960 min). In relation to the three formal mindfulness practices (body scan, mindful movement, and sitting meditation), six studies (11%) reported the inclusion of sitting meditation alone, 12 studies (21%) included two MBP practices, and the remaining 38 studies (68%) included all three practices. Two-thirds (*k* = 37) of studies reported details of the frequency and duration of assigned home mindfulness practice. The suggested frequency ranged from five to seven days a week (median seven days), while the suggested daily duration ranged from five to 60 min (median 20 min).

Forty-two studies (75%) reported quantitative (e.g. amount of time accrued in mindfulness teaching or practice) and qualitative (e.g. certification status) information about the MBP facilitators’ credentials. Broadly, thirty-three studies (59%) described the facilitator as being a mindfulness teacher/instructor (*k* = 22 (39%) explicitly stating that the facilitator had completed MBP teacher training). The remaining nine studies (16%) simply described the facilitator as being a clinician, a mindfulness practitioner, or an individual with limited mindfulness teaching experience.

Twenty-five studies (45%) reported adherence data for the MBP. Across these studies, the mean proportion of sessions attended ranged between 39 and 100% (median 87%). Some studies, however, excluded dropouts from the reported adherence data. If dropouts were accounted for, adherence would be expected to decrease. Home practice adherence data were available for 16 studies (29%). Eleven studies (20%) reported home practice data as the mean reported duration of practice; these figures ranged from 36 to 100% (median 82%) of the amount assigned in the MBP. Five studies (9%) reported home practice data as the mean percentage of assigned home sessions completed; these ranged from 57 to 100% (median 84%).

Forty-eight studies (86%) included a single comparator group, while eight studies (14%) included more than one comparator. Of the latter, five studies (9%) included one active and one inactive comparator. In the main analyses, the MBP was compared against the active comparator intervention for these studies, while both comparisons were included in subgroup analyses presenting data separately for active and inactive comparators. Two (4%) of the studies with more than one comparator included two MBP arms and a single inactive arm. For these studies, we selected the four-week modified MBP arm (Zanesco et al., 2019) and the modified MBP arm (Lymeus et al., 2016) for all analyses, and compared these against the inactive comparator. The final study (Bowden et al., 2012) included two active comparator interventions: Body and Brain Training, and Iyengar Yoga. The MBP was compared against the yoga group in all analyses, as yoga is a better-researched intervention, and may positively impact cognition (Gothe & McAuley, 2015).

Considering only the ‘main’ comparator group (i.e. that used in the main analyses), the review included 30 inactively-controlled studies (54%), the majority of which (*k* = 20; 36%) used a waitlist. Of the ten (18%) which did not, five (9%) recruited clinical samples and offered the control group treatment as usual, and five studies (9%) recruited non-clinical samples and offered no intervention to the control group. Twenty-six studies (46%) used active comparator interventions, all of which were group-based. These comprised health enhancement/education programs (*k* = 7; 13%), psychoeducation interventions (*k* = 7; 13%), or relaxation interventions (*k* = 4; 7%). The remaining studies utilized cognitive training (*k* = 2; 4%); nutrition (*k* = 2; 4%); reading (*k* = 2; 4%); yoga (*k* = 1; 1%); or social support (*k* = 1; 1%) interventions. All except one of the active comparator interventions (*k* = 25; 45%) matched the relevant MBP for number of sessions; all active comparators matched MBP session duration. Nineteen studies (34%) provided clear information about the provision of homework in both the MBP and active comparator arms. The MBP and active comparator were equivalent for homework provision in 17 studies (30%), while two studies (4%) assigned homework to MBP participants only. Twelve actively-controlled studies (21%) provided clear information about the number of retreats in the MBP and comparator arms. Ten studies (18%) featured an active comparator which matched the MBP for number of retreats (including six studies without retreats). The remaining two studies (4%) featured an MBP retreat but no comparator retreat. Where ascertainable, session number and duration, homework, and retreat provision were thus approximately matched between MBPs and active comparator interventions.

### Risk of Bias

**Fig. 2:**
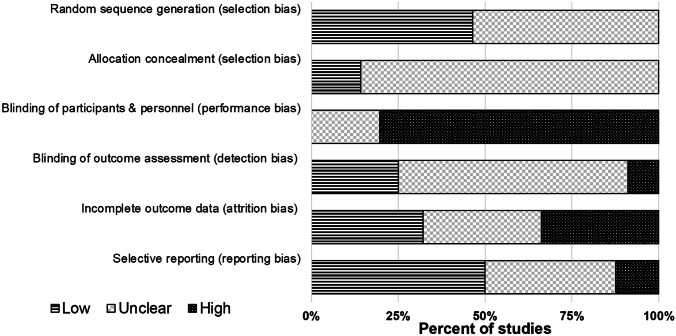
Cochrane Risk of Bias Graph. The risk of bias graph presents ratings for all 56 studies included in the systematic review

The methodological quality of the studies reported varied significantly (see Fig. 2). ‘Random sequence generation’ bias ratings were split between ‘Low’ (*k* = 26; 46%) and ‘Unclear’ (*k* = 30; 54%). The majority of studies (*k* = 48; 86%) did not report enough information to assess the potential for ‘Allocation concealment’ bias and were thus rated ‘Unclear’. A large proportion of studies (*k* = 45; 80%) were rated as being at ‘High’ risk of bias for ‘Blinding of participants and personnel’, given the inherent difficulties in achieving this in nonpharmacological RCTs. The ‘Blinding of outcome assessment’ domain saw fourteen studies (25%) rated as being at ‘Low’ risk, and five (9%) studies rated as ‘High’ risk (e.g. for not blinding psychometrists), although the majority of studies (*k* = 37; 66%) were ‘Unclear’. Ratings for ‘Incomplete outcome data’ bias were split between ‘Low’ (*k* = 18; 32%), ‘High’ (*k* = 19; 34%) and ‘Unclear’ (*k* = 19; 34%). In this category, ‘High’ bias ratings reflected high overall attrition (≥ 30%) and/or use of per-protocol analyses. Ratings for bias associated with ‘Selective reporting’ were mainly ‘Low’ (*k* = 28; 50%) or ‘Unclear’ (*k* = 21; 38%), although seven studies (13%) were considered to be at ‘High’ risk (e.g. for not reporting results for a cognitive test mentioned in the study Method and/or trial registration). Across each of the six risk of bias domains, the median proportion (range) of studies rated as ‘Low’ was 29% (0 to 50%); ‘High’, 11% (0 to 80%); and ‘Unclear’, 46% (20 to 86%).

### Publication Bias

Robust variance estimation meta-regression found no significant association between the standard error of effect sizes and effect sizes themselves – either overall, or for actively- or inactively-controlled studies separately (see supplementary Table S4). We therefore did not identify evidence of bias stemming from underrepresentation of small sample size studies with null or negative findings. Examining the intercept (interpretable as the estimate for a hypothetical, infinitely large study) for each of these meta-regression models revealed the following values [95% confidence intervals]: all studies 0.33; [-0.01, 0.68]; actively-controlled 0.10; [-0.51, 0.72]; and inactively-controlled 0.46; [0.10, 0.82]. In each of these models, the coefficient for the SE meta-regressor was negative, thus explaining how adjusting for small study effects resulted in larger effect size estimates compared to the unadjusted meta-analyses (see next section).

### Quantitative Synthesis of Results

Forty-six studies (82%) reported cognitive outcome data amenable to meta-analysis (or the authors provided these on request). The remaining ten studies (18%) were excluded from meta-analyses for the following reasons: only reporting non-summary scores for laboratory tests (*k* = 7; 13%); not reporting sufficient data to calculate effect sizes (*k* = 2; 4%); or not reporting cognitive test data (*k* = 1; 2%). No included study reported data for the language or reasoning cognitive domains. Available effect sizes (*n*; %) thus measured executive function (63; 35%), attention (53; 29%), declarative memory (49; 27%), cognitive aging (7; 4%), construction (5; 3%), or visual perception (3; 2%). No study administered a non-visual measure of perception. See supplementary Table S3 for a complete list of analyzed cognitive test measures and metrics.

### Primary Analysis

The initial meta-analysis pooled all 180 effect sizes from 46 studies. The pooled effect size, collapsing across cognitive domains, significantly favored MBPs over comparators (*g* = 0.23; [0.01, 0.46]). This analysis included the trial reported by Rothschild et al. (2017), which compared a 72-session MBP to an inactive comparator, and reported an unusually large effect size (*g* = 5.2) for the digit symbol substitution test (the only eligible measure). Given that both the number of MBP sessions (median number of sessions was eight) and effect size were outliers, this study was removed and the analyses repeated. Following the removal of this study, the updated meta-analytic estimate continued to favor MBPs over comparators but was somewhat attenuated (*g* = 0.15; [0.05, 0.24]; see Table 2). The *I*^*2*^ statistics for the initial and updated model were 80% and 20%, respectively, suggesting that the study by Rothschild et al. (2017) was indeed a statistical/methodological outlier. We thus denoted the updated meta-analysis the ‘final’ model, with all analyses reported hereafter comprising 179 effect sizes from 45 studies (*n* = 2,238).

**Table 2:** Meta-analyses comparing MBPs to comparators for all cognitive domains and subdomains (both combined and separately)

Domain	Subdomain	*K* (*N* ES)	ES *(g)*	95% CI	*df*	*p*-value	*Tau*^*2*^	*I*^*2*^
All domains^**#**^	NA	45 (179)	**0.15**	[0.05, 0.24]	36	0.004	0.02	20.08
Executive function	All combined	29 (63)	**0.15**	[0.02, 0.27]	23	0.022	0.02	18.74
	Cognitive flexibility	10 (19)	0.08	[-0.20, 0.35]	8	0.549	0.06	40.61
	Working memory	13 (21)	**0.23**	[0.11, 0.36]	9	0.002	0.00	0.00
	Inhibition	17 (23)	0.10	[-0.06, 0.27]	14	0.205	0.02	18.54
Attention	All combined	22 (52)	0.12	[-0.02, 0.26]	18	0.096	0.03	21.79
	Alerting	22 (46)	0.08	[-0.07, 0.24]	18	0.265	0.02	18.71
	Orienting	5 (6)	0.15	[-0.41, 0.71]	3*	*	0.09	45.79
Declarative memory	All combined	14 (49)	0.14	[-0.02, 0.30]	9	0.076	0.00	0.00
	Episodic memory	9 (41)	0.10	[-0.11, 0.31]	7	0.285	0.01	11.66
	Short-term memory	7 (8)	0.16	[-0.13, 0.45]	4	0.208	0.00	0.00
Cognitive aging	NA	6 (7)	0.07	[-0.22, 0.36]	4	0.530	0.00	0.00
Construction	NA	5 (5)	-0.01	[-0.25, 0.23]	3*	*	0.00	0.00
Visual perception	NA	2 (3)	0.33	[-2.55, 3.22]	1*	*	0.03	20.28

Effects in **bold** reached statistical significance (*p* < 0.05)

*MBP* Mindfulness-based program, *K* Number of studies, *ES* Effect size, *g* Hedges’ standardized mean difference (positive values imply improvement), *CI* Confidence interval, *df* Degrees of freedom, *NA* Not applicable (no subdomains were specified)

^#^Excluding the study by Rothschild et al. (2017); *Where *df* < 4, *p*-values are unreliable, and are thus not reported here

**Table 3:** Subgroup analyses of key moderators (sample, intervention, and methodological characteristics).

Category	Subcategory	*K* (*N* ES)	ES *(g)*	95% CI	*df*	*p*-value	*Tau*^*2*^	*I*^*2*^
Age group	Adults (< 60 years)	30 (115)	0.11	[-0.01, 0.24]	26	0.079	0.03	23.81
	Older adults (≥ 60 years)	15 (64)	**0.21**	[0.04, 0.38]	9	0.020	0.01	10.50
Clinical status	Clinical	18 (76)	0.09	[-0.06, 0.24]	14	0.208	0.01	7.81
	Non-clinical	27 (103)	**0.18**	[0.05, 0.31]	22	0.010	0.03	26.25
MBP type	Unmodified MBSR/MBCT	12 (39)	0.04	[-0.22, 0.30]	10	0.720	0.09	53.21
	Modified MBP	23 (102)	**0.15**	[0.03, 0.26]	17	0.017	0.00	0.00
	Generic MBP	10 (38)	**0.26**	[0.05, 0.47]	8	0.022	0.04	30.66
Comparator type	Active	22 (84)	0.07	[-0.04, 0.19]	18	0.188	0.00	0.00
	Inactive*	28 (120)	**0.20**	[0.06, 0.33]	22	0.006	0.04	27.41
Risk of bias^#^	Lower	12 (38)	0.18	[-0.01, 0.38]	7	0.065	0.01	6.86
	Higher	33 (141)	**0.14**	[0.01, 0.26]	28	0.030	0.03	25.33
Trial registration	Registered	15 (57)	**0.23**	[0.07, 0.39]	11	0.008	0.02	18.15
	Not registered	30 (122)	0.09	[-0.03, 0.22]	25	0.146	0.02	17.34

Effects in **bold** reached statistical significance (*p* < 0.05)

*K* Number of studies, *ES* Effect size, *g* Hedges’ standardized mean difference (positive values imply improvement), *CI* Confidence interval, *df* Degrees of freedom, *MBP* Mindfulness-based program, *MBSR* Mindfulness-Based Stress Reduction, *MBCT* Mindfulness-Based Cognitive Therapy

*Includes an additional inactive arm from each of the five studies which featured both active and inactive comparators; ^#^Here, studies with ≥ 3 ‘Low’ ratings (of a maximum of six) were considered ‘Lower’ risk, otherwise ‘Higher’ risk

### Meta-regression

Univariable meta-regressions evaluated the following candidate moderators in the final meta-analytic dataset: type of comparator; age group; clinical status; type of MBP; number of formal mindfulness practices included; whether a retreat was included; number of MBP sessions; frequency of MBP sessions; and duration of MBP sessions. Each moderator was first individually meta-regressed on effect size, and none were significant (all *p*s > 0.08). Whilst no moderators were significant, those effecting the greatest reduction in the *I*^*2*^ statistic (relative to the final meta-analysis) were type of comparator (*I*^*2*^ reduced by 4.0 percentage points); MBP session duration (*I*^*2*^ reduced by 3.7 percentage points); and MBP session frequency (*I*^*2*^ reduced by 1.8 percentage points). All moderators were then simultaneously included in a meta-regression, and none emerged as significant (all *p*s > 0.25; see supplementary Table S5). These analyses were repeated substituting continuous age in years for age groups; this produced the same pattern of findings as described for the univariable age and multiple meta-regressions.

### Subgroup Analyses

#### Cognitive Domains and Subdomains

Outcomes were subdivided according to the cognitive domain they primarily represented. MBPs significantly outperformed comparators for executive function (*k* = 29; *g* = 0.15; [0.02, 0.27]; see Table 2). MBPs did not significantly outperform comparators for attention, declarative memory, nor cognitive aging. There were insufficient data to yield reliable *p*-values for the visual perception or construction domains (see supplementary materials for a narrative review of those results).

Executive function, attention and declarative memory were further divided into subdomains; these were also evaluated separately (see Table 2). The only subdomain for which MBPs significantly outperformed comparators was for the working memory subdomain of executive function (*k* = 13; *g* = 0.23; [0.11, 0.36]).

Six studies reported data from the Attention Network Test. In contrast to the variability of measures included in other analyses, these data provided the opportunity to examine effects on a single measure. MBPs did not outperform comparators for any of the network scores (i.e. alerting, orienting and executive; see supplementary Table S6).

#### Clinical Status and Age Group

In studies including non-clinical samples, MBPs significantly outperformed comparators (*k* = 27; *g* = 0.18; [0.05, 0.31]). In studies of clinical samples, MBPs did not significantly outperform comparators (*k* = 18; *g* = 0.09; [-0.06, 0.24]; see Table 3).

MBPs did not outperform comparators in studies of adult (< 60 years) samples (*k* = 30; *g* = 0.11; [-0.01, 0.24]). MBPs significantly outperformed comparators for older adult (≥ 60 years) samples (*k* = 15; *g* = 0.21; [0.04, 0.38]; see Table 3). Following this finding, exploratory analyses evaluated separate cognitive domains for older adults; MBPs significantly outperformed comparators for the executive function domain only (*k* = 8; *g* = 0.27; [0.05, 0.50]; see supplementary Table S7). We also conducted separate, exploratory subgroup analyses of clinical and non-clinical samples of older adults; neither subgroup exhibited significant effects, although the clinical analysis did not return a reliable *p*-value, and the lower confidence bound for the non-clinical estimate approached zero (*g* = 0.25; [-0.01, 0.51]; see supplementary Table S8).

#### Comparator and MBP Type

MBPs did not significantly outperform active comparator interventions (*k* = 22; *g* = 0.07; [-0.04, 0.19]). In contrast, MBPs significantly outperformed inactive comparators (*k* = 28; *g* = 0.20; [0.06, 0.33]; see Table 3). Across these subgroups, the total number of ‘studies’ exceeded the total for other analyses (i.e. 50 versus 45), reflecting that some studies included both an active and an inactive comparator.

Unmodified MBSR/MBCT did not significantly outperform comparators (*k* = 12; *g* = 0.04; [-0.22, 0.30]). Modified MBPs outperformed comparators (*k* = 23; *g* = 0.15; [0.03, 0.26]), as did generic MBPs (*k* = 10; *g* = 0.26; [0.05, 0.47]; see Table 3). We conducted additional, exploratory subgroup analyses, seeking to better understand these results. No MBP type outperformed comparators when actively- and inactively-controlled studies were analyzed separately (see supplementary Table S9). However, subgrouping each type of MBP for clinical and non-clinical samples separately indicated that modified MBPs outperformed comparators in clinical samples, and generic MBPs outperformed comparators in non-clinical samples (see supplementary Table S10). Of note, nine of the ten studies utilizing generic MBPs were conducted in non-clinical samples, which may partially explain the comparatively large ‘main’ estimate for this MBP type.

#### Risk of Bias

We conducted unplanned subgroup analyses of studies which received a ‘Low’ risk of bias rating in at least three of six domains (defining this subgroup as ‘Lower’ risk), as well as for studies with fewer than three ‘Low’ ratings (defined as ‘Higher risk’), in order to establish whether risk of bias was associated with effect size. We acknowledge that requiring a minimum of only three ‘Low’ ratings to qualify a study as being at lower overall risk is perhaps overinclusive. However, only 12 studies satisfied this criterion, and the relevant threshold was thus motivated by statistical expediency. MBPs did not significantly outperform comparators in studies at lower risk of bias (*k* = 12; *g* = 0.18; [-0.01, 0.38]; see Table 3). In contrast, MBPs significantly outperformed comparators in studies at higher risk of bias (*k* = 33; *g* = 0.14; [0.01, 0.26]). Whilst this might appear to support the notion that higher risk of bias studies were associated with greater effects, the estimates were of a similar magnitude, and in both cases the lower confidence bound approached zero, implying that the significance (or lack thereof) of these analyses might exaggerate the difference between subgroups. Interestingly, the *I*^*2*^ statistic was nominally smaller for studies at lower (7%) versus higher (25%) risk of bias.

Whilst trial registration status does not explicitly inform Cochrane risk of bias ratings, registered studies are considered to be at lower risk of reporting bias. It was recently recommended that meta-analysts thus conduct separate subgroup analyses of registered and unregistered trials, to ascertain whether trial registration status moderates effect sizes (Trinquart et al., 2018). We thus performed the recommended (unplanned) subgroup analyses and found that MBPs significantly outperformed comparators in registered studies (*k* = 15; *g* = 0.23; [0.07, 0.39]), but that there was no difference in unregistered studies (*k* = 30; *g* = 0.09; [-0.03, 0.22]; see Table 3).

## Discussion

We identified 56 randomized MBP studies which measured objective cognition, of which approximately 30% comprised older adult samples (≥ 60 years), and 70% adult samples (< 60 years). Around forty percent of studies recruited participants from clinical populations, primarily comprising individuals with neurocognitive, psychiatric, or neurological disorders; the remaining 60% included non-clinical samples. About a quarter of studies used unmodified MBSR/MBCT, half featured modified MBPs, and the remainder used generic MBPs. The split between actively- and inactively-controlled designs was approximately equal.

Forty-five studies were included in the final meta-analysis. The summary effect (pooling data across cognitive domains) significantly favored MBPs over comparators and was small in magnitude (*g* = 0.15; [0.05, 0.24]; see Table 2). Subgroup meta-analyses identified a significant effect for executive function (*g* = 0.15; [0.02, 0.27]), but not attention, declarative memory or cognitive aging. There were insufficient data to meta-analyze the construction or visual perception domains, though no original studies reported MBP effects for these outcomes. Investigating the subdomains of executive function, MBPs conferred a significant benefit to working memory (*g* = 0.23; [0.11, 0.36]), but not cognitive flexibility or inhibition.

### Cognitive Domains and Subdomains

The theorized cognitive benefits of MBP participation are domain-specific (Hölzel et al., 2011; Lutz et al., 2008; Shapiro et al., 2006; Vago & Silbersweig, 2012), and strongly implicate the domains of attention and executive function. We conducted subgroup analyses of separate cognitive domains and subdomains, enabling observed effects to be compared to theorized gains. It remains important for the reader to hold in mind that, whilst MBPs outperformed inactive comparators, they did not outperform active comparator interventions. Significant effects could thus relate to aspects of interventions which are common to both MBPs and the presently included active comparators (e.g. therapeutic alliance, social stimulation, and/or treatment expectancy).

### Executive Function

There was strong evidence for a small effect (*g* = 0.15; [0.02, 0.27]) of MBP participation on executive function. This is in-keeping with a recent meta-analysis of mindfulness meditation and executive function (Cásedas et al., 2020). However, earlier systematic reviews did not identify an effect in this domain (Chiesa et al., 2011; Lao et al., 2016), perhaps due to the comparatively small evidence base previously available. In contrast, both the current meta-analysis and all three prior reviews identified improvements to working memory (here operationalized as a subdomain of executive function). A recent opinion paper discussing the relationship between working memory and mindfulness practice noted the latter ‘may repeatedly require selective and reflective attentional engagement, disengagement, maintenance, and monitoring […] these processes are also necessary to successfully maintain and manipulate information in working memory’ (Jha et al. (2019), p.274). A proposed explanation for the present effect could, therefore, be the engagement of cognitive processes involved in working memory during mindfulness practice.

Mindfulness frameworks suggest that practicing focused attention meditation might confer benefit to the cognitive flexibility subdomain of executive function, given the emphasis on noticing mind wandering and refocusing attention (Gallant, 2016; Hölzel et al., 2011; Lutz et al., 2008; Shapiro et al., 2006; Vago & Silbersweig, 2012). In-keeping with an earlier meta-analysis (Cásedas et al., 2020), we did not identify a significant effect on cognitive flexibility. A possible explanation for this involves reframing sitting meditation as a unitary attention task in the presence of off-task distractors (e.g. mind wandering), whereas cognitive flexibility may be considered the ability to volitionally shift between tasks.

Neurocognitive frameworks of mindfulness hypothesize that practice should lead to improved attentional, emotional and behavioral self-regulation (Shapiro et al., 2006). Vago and Silbersweig (2012) theorize that the mechanism underlying this is the development of inhibitory control processes, which are thought to be recruited during mindfulness practice in support of attentional regulation. In contrast to Cásedas et al. (2020), the present review did not observe a significant benefit to inhibition following MBP participation. It remains pertinent, however, to consider the limitations of tasks typically used by the presently-included studies to measure inhibition. For example, discussing the Stroop test, Vago et al. (2019) noted that studies have demonstrated a lack of convergent validity between different formats, as well as poor test–retest and internal reliability. Other measures of inhibition included here also have poor test–retest reliability, for example the Attention Network Test (see next section).

### Attention

Perhaps surprisingly, we did not identify an effect of MBP participation on attention. It is first worth noting that relevant mindfulness frameworks emphasize the role of attentional regulation above attention itself (Lutz et al., 2008). The processes involved in attentional regulation are considered to be delineable from those involved in basic attention, and might share more in common with executive function (Petersen & Posner, 2012). Moreover, we encountered significant variability amongst tests of attention. These could broadly be grouped into pen-and-paper tests (e.g. WAIS digit symbol coding, cancelation tests) and computerized tests (e.g. Attention Network Test, Continuous Performance Test). Notably, the test–retest reliabilities (reported as intraclass correlations; ICCs) of currently-included tests of both formats vary considerably. One guideline (Koo & Li, 2016) suggests the following rubric for interpreting ICC values: poor (< 0.50), moderate (0.50 to 0.75), good (0.75 to 0.90), and excellent (> 0.90). The attentional outcomes administered by the present studies thus include measures with good (e.g. WAIS digit symbol coding; Lezak (2012)), moderate (e.g. AX-Continuous Performance Test; Cooper et al. (2017)), and poor reliability (e.g. Attention Network Test; Enkavi et al. (2019)). Further research utilizing attentional measures with good or excellent reliability will enable more confident conclusions regarding effects in this domain.

### Declarative Memory

The majority of declarative memory measures utilized verbal stimuli (e.g. auditory verbal learning tests, WAIS logical memory test). While the contemporary discourse surrounding MBPs emphasizes attentional and emotional regulation, classical Buddhist texts also equate mindfulness with the ability to accurately recall prior events (see Brown et al. (2016)). Presently, the effects for the declarative memory domain and subdomains (i.e. episodic, and short-term memory) were not significant. Previous reviews (Chiesa et al., 2011; Lao et al., 2016) did not discuss effects on declarative memory as available data were sparse. However, both reviews reported an effect on memory specificity (the preferential recall of specific versus generalized autobiographical memories), the dysfunction of which has been implicated in affective disorders (Williams et al., 2007). Memory specificity outcomes were not included in this review, precluding comparisons with previous syntheses.

### Cognitive Aging

Six studies (four representing patients with neurocognitive disorders, and two of non-clinical samples) included measures of cognitive aging. MBPs did not outperform comparators for this domain. Four studies administered the MMSE (Folstein et al., 1975), a very brief screening instrument for cognitive impairment. The MMSE does not include any executive function subitems, here the only domain to improve separately. Furthermore, the MMSE is not recommended for use in interventional studies due to floor and ceiling effects and is not sensitive to changes in persons without dementia (Posner et al., 2017). Further studies utilizing more in-depth measures of cognitive aging are thus required for a more rigorous evaluation of this domain.

### Age Group

#### Older Adults

Whilst earlier reviews summarized MBP effects on cognition in older adults (Berk et al., 2017; Fountain-Zaragoza & Prakash, 2017; Hazlett-Stevens et al., 2019), none were explicitly systematic (though see Gard et al. (2014) for a systematic review of mindfulness *meditation*). The reviews found mixed evidence for cognitive effects in elders, and concurred that additional high-quality studies were needed to support a more confident conclusion. The literature has since grown, with this synthesis being the first to systematically review and meta-analyze data from MBP studies of older adults (here a subgroup analysis). Across 15 studies of older adults (≥ 60 years; *n* = 860), the pooled effect across cognitive domains favored MBPs over control conditions (*g* = 0.21; [0.04, 0.38]). Considering cognitive domains separately for these studies, MBPs did not outperform comparators on attention, declarative memory, or cognitive aging. However, the pooled effect for executive function (*g* = 0.27; [0.05, 0.50]) favored MBPs, with this effect being nominally larger than the equivalent effect for adults and older adults combined (*g* = 0.15; [0.02, 0.27]). A recent analysis of over 470,000 individuals from the UK Biobank identified an average 7.8% decline in performance on the Trail-making test part B (a test of executive function) for each additional age group (defined as five-year intervals) beyond age 45 (Cornelis et al., 2019). Executive function thus declines with age, with the evidence presented here suggesting MBPs improve executive function in older adults; MBPs may thus be of particular value to older adults for supporting the partial restoration of function in this domain.

#### Adults

In contrast to older adults, the subgroup analysis of adults (< 60 years; *k* = 30; *n* = 1,378) including data from all cognitive domains did not identify a difference between MBPs and comparators (*g* = 0.11; [-0.01, 0.24]). One interpretation of this apparent age-specific effect is that MBPs might be helpful for restoring cognitive abilities tending to decline over age 60 (see above). This interpretation is based on the intuition that restoring cognitive abilities to a previous state might be more easily achieved than improving abilities beyond the developmental peak.

### Clinical Status

We divided studies into subgroups based on the clinical status of participants. Eighteen studies recruited patients with clinical diagnoses (predominantly comprising samples with neurocognitive, psychiatric, or neurological disorders). In clinical samples, MBPs did not improve overall cognitive function relative to comparators. In contrast, across the 27 studies of non-clinical samples, MBPs significantly outperformed comparators across the combined analysis of cognitive domains (*g* = 0.18; [0.05, 0.31]). Potential explanations for the difference in effects between clinical and non-clinical samples include that relatively greater variability between and within clinical samples may have obscured effects; that some clinical conditions might interfere with the ability to intensively engage with mindfulness practices; and that some clinical studies used insensitive test measures (e.g. the MMSE).

### Risk of Bias Between and Within Studies

We did not identify evidence of an association between effect sizes and their standard errors, suggesting that small study effects are unlikely to account for the present results. Considering studies at the individual level, the median proportion of ‘Low’ ratings made across the six Cochrane risk of bias domains was 29%. A substantial portion of included studies did not satisfactorily report the elements required for comprehensive risk of bias assessment (the median proportion of ‘Unclear’ judgments was 46%). The rate of trial registration was relatively low amongst current studies (33%), a factor putatively linked to inflated meta-analytic estimates in the psychological literature (Kvarven et al., 2020). However, in subgroup analyses we identified a significant effect for registered but *not unregistered* studies, implying that the relatively large proportion of unregistered trials has not upwardly biased current effect estimates. Nevertheless, the frequently unclear risk of bias highlights the uncertain methodological quality of some of the evidence on which this review is based.

### Complementary Lines of Evidence

During our systematic review of the extant literature, we identified a number of studies that did not meet the inclusion criterion of randomizing participants or did not feature an MBP fully satisfying the present criteria yet demonstrated a significant impact on cognition. Indeed, over fifty studies have employed other study designs to investigate the impact of mindfulness training on cognition; these should be taken into careful consideration when making any conclusions concerning the effects of mindfulness practice. Chiesa et al. (2011) undertook an early review of studies investigating the effect of different forms of meditation on cognition using cross-sectional designs, or other secularized forms of training that involve some overlap with MBPs such as Acceptance and Commitment Therapy (Chiesa et al., 2011). For example, Jha et al. (2007) compared participants following an 8-week MBP; experienced meditators following an intensive retreat; and an inactive comparator group. Jha et al. (2007) demonstrated uniquely improved orienting on the Attention Network Test in the MBP group, and improved alerting in the retreat group, relative to inactive controls (Jha et al., 2007). Numerous non-RCT studies in clinical and non-clinical populations have found MBPs outperform inactive comparators on the Continuous Performance Test (Bueno et al., 2015); attentional bias using affective priming (De Raedt et al., 2012) or dot-probe tasks (Garland, 2011; Garland et al., 2013; Vago & Nakamura, 2011); a task-switching paradigm (Greenberg et al., 2017); and Stroop test (Basso et al., 2019). Other studies have shown that mindfulness training with variable duration and intensity have significant effects on cognitive performance in non-clinical samples. For example, mindfulness training with as few as four 20-min sessions (Zeidan et al., 2010) improved performance on processing speed, verbal fluency and working memory versus an active comparator intervention. Other studies have compared mindful state induction techniques seven to 20 min in length against active controls, with cognitive testing immediately following the practice session. These studies demonstrated improved inattentional blindness (Schofield et al., 2015); alerting (Polak, 2009); executive attention on a flanker task (Norris et al., 2018); attentional blink (Colzato, et al., 2015a, 2015b); cognitive control on a Simon task (Colzato et al., 2015a, 2015b); and cognitive performance on a Stroop test (Wenk-Sormaz, 2005). Studies in meditation-naïve healthy populations that use intensive retreat formats up to three months in duration requiring 10–12 h of daily practice have also shown benefits on cognitive outcomes (Sahdra et al., 2011; Trautwein et al., 2020). Some studies demonstrating improved cognition have modified MBPs to address the needs of a specific population, like the Mindfulness-based mind fitness training course developed to address the specific needs of the military (Jha et al., 2010, 2015) or the Mindfulness-oriented recovery enhancement program which focuses on addiction, opioid-dependent and chronic pain populations (Garland & Howard, 2013; Garland et al., 2017). A number of non-RCT studies have now shown improvements in working memory using the operation span task (Jha et al., 2010), or a lack of degradation of sustained attention, inhibitory control capacity and working memory in military cohorts following Mindfulness-based mind fitness training (Jha et al., 2015, 2017, 2020). Participants randomized to Mindfulness-oriented recovery enhancement exhibited significantly reduced attentional bias in comparison to active controls (Garland & Howard, 2013; Garland et al., 2017). Furthermore, reductions in attentional bias predicted improvements in opioid use at three month follow-up (Garland et al., 2017). Cross-sectional studies of meditators versus non-meditators show enhanced attention and working memory in meditators on the Stroop test (Fabio & Towey, 2018; Moore & Malinowski, 2009); attentional blink (Fabio & Towey, 2018; Slagter et al., 2007); n-back (Fabio & Towey, 2018); and rapid visual information processing task (Pagnoni & Cekic, 2007). Other studies have observed improved cognitive performance in individuals who score higher on trait mindfulness scales (Schmertz et al., 2009), suggesting dispositional differences. Thus, in addition to the current findings, one must consider the existing literature at the granular level to build a well-informed overview.

### Strengths of the Review

This evidence synthesis has a number of clear strengths. Previous reviews did not search the literature systematically, or combined data from both longitudinal and cross-sectional studies (Berk et al., 2017; Chiesa et al., 2011; Fountain-Zaragoza & Prakash, 2017). In contrast, we conducted a systematic literature search; solely included randomized studies; and performed screening, bias rating, and data extraction in duplicate. Moreover, we only included objective behavioral assessments of cognition, thereby circumventing problems associated with self-report measures (Van Dam et al., 2018). Perhaps most significantly, to our knowledge this is the first meta-analysis of general cognitive outcomes (i.e. spanning multiple domains) from randomized MBP studies. The rapidly-expanding MBP literature constitutes a mosaic of significant and non-significant effects across various cognitive domains, the complexity of which prevents interested readers from gaining an intuitive sense of the aggregate effects. The present synthesis thus represents a genuine advance for the field, as it evaluates the effects of MBPs on separate cognitive domains, and also evaluates key moderators such as age, clinical status, and MBP type. Moreover, the robust variance estimation meta-analytic approach was specifically selected to accommodate multiple effect sizes within studies, which avoided the averaging or simplifying of data.

### Limitations of the Review

In spite of the clear strengths, limitations remain. Perhaps the most general limitation relates to the variability amongst study populations, MBPs, comparators and outcomes. In particular, the studies comprising the current ‘clinical’ dataset recruited from a range of populations, including individuals with neurocognitive, psychiatric or neurological disorders. Moreover, we were unable to evaluate the potentially moderating effects of some MBP characteristics (e.g. the amount of teaching of mindfulness theory, the amount of ‘informal’ home mindfulness practice assigned or completed). These characteristics varied between studies and may contribute to unexplained variability in some statistical models. Half of the studies were actively-controlled, with these featuring a variety of comparator interventions, controlling for different aspects of MBPs. Lastly, the included cognitive outcomes spanned multiple domains; represented both pen-and-paper and computerized paradigms; and were scored and reported in a variety of ways.

We sought to accommodate these sources of variability using subgroup analyses and meta-regression. Although meta-regression did not identify significant moderators, subgroup analyses returned a mixture of significant and non-significant effects. It remains important to acknowledge the limitations of subgroup analyses and meta-regression, given that the former is prone to confounding (Spineli & Pandis, 2020), and the latter low statistical power (Hempel et al., 2013). Nevertheless, the continued publication of MBP studies will enable more powerful meta-regression analyses in future syntheses.

In the primary meta-analysis, we combined outcomes across cognitive domains. A previous meta-analysis corroborated the view that tests generally measure more than one domain (Agelink van Rentergem et al., 2020), providing empirical support for the present all-domain analyses. Moreover, evidence syntheses of other nonpharmacological interventions also included pooled analyses (Mewborn et al., 2017; Sherman et al., 2017). Nevertheless, this approach does not yield a true measure of overall cognitive function, and thus should be interpreted with a degree of caution. Lastly, the methodological rigor of individual studies was varied, with the most frequent finding being ‘Unclear’ risk of bias (only a minority of judgments yielded ‘Low’ risk ratings).

### Implications of the Review

Firstly, it is worth acknowledging that the small magnitude of the primary meta-analytic estimate (*g* = 0.15; [0.05, 0.24]). We encourage the interpretation of this with reference to the length of the included MBPs, which spanned six to 12 weeks. It remains possible that interventions delivered over a longer period, or in intensive retreat settings, might confer larger gains. The degree to which participants engage with mindfulness practices post-intervention may also contribute to longer-term cognitive effects. Evaluating this requires post-intervention follow-up assessments, which were generally lacking amongst present studies; where practicable, the inclusion of follow-up timepoints in future MBP trials will clarify the putative role of ongoing mindfulness practice.

Considering a general implication, we echo Van Dam et al. (2018) in encouraging methodological improvements in MBP research. Investigators are encouraged to compare MBPs to active comparators (either alone or in addition to inactive comparators, which may help to parse out practice effects). The increased use of active comparators will better enable specific and non-specific intervention effects to be disentangled. We recommend that studies be preregistered; that strict randomization procedures are implemented and reported; and that data are analyzed using intention-to-treat. We also encourage greater adoption of standardized cognitive measures – particularly those pertaining to executive function and working memory (e.g. the NIH Toolbox Cognition Battery, NIH EXAMINER). This promises to improve the validity, reliability and comparability of studies (Vago et al., 2019). Only a single study (Lebares et al., 2019) utilized such a test measure in the present review.

MBP studies combining both neurophysiological and objective cognitive measures are encouraged; electroencephalography (EEG) is particularly well-suited to the study of time-sensitive executive function processes (Falkenstein et al., 1999; Van Veen & Carter, 2002). Indeed, error processing and performance monitoring have already been measured via EEG in some MBP trials (Schoenberg & Speckens, 2014; Schoenberg et al., 2014). Future research will likely delineate the specific MBP components driving cognitive benefits for specific populations and optimize delivery accordingly. Optimized MBPs will have the added benefit of reducing the variability that abounds in the way mindfulness is currently taught across different sectors of society. Whilst variability amongst MBPs constitutes a current limitation, in one respect, future reviews may consider encompassing greater variability. Namely, there is increasing interest in the digital delivery (e.g. via computer or smartphone) of both nonpharmacological interventions in general, and MBPs specifically. Future syntheses may seek to focus on digitally-delivered MBPs, or even to compare different delivery modes. Variability in participant motivation, and the degree to which it may moderate effects in MBP studies, is also a relatively under-researched area. Whilst participant adherence may be used as a proxy for motivation, less than half of studies included here reported adherence data, precluding our evaluation of this putative moderator.

The present finding that MBPs improve cognition in older adults (≥ 60 years) but not adults (< 60 years) indicates that these interventions may help guard against cognitive decline, rather than improving cognitive skills more generally. Further studies in older participants utilizing follow-up assessments will enable this implication to be explored more fully.

A further consideration relates to the potential transfer of cognitive training effects. Interventions may improve performance on (i) the trained task; (ii) on closely related tasks; (iii) on distantly related tasks; and/or (iv) everyday cognitive functioning. Achieving improved performance on the trained task is considered easier than for untrained/distal tasks (Simons et al., 2016). Returning to our present focus, mindfulness practices do not, ostensibly, share a great deal in common with cognitive test measures. In particular, the primary foci of mindfulness practices are subjective and internal (e.g. thoughts, emotions). In contrast, cognitive measures exclusively utilize objective visual or auditory stimuli. In spite of these differences, the present review identified MBP effects on all-domain cognition, as well as for executive function and working memory. This suggests that MBP training effects may partially transfer to cognitive activities beyond those directly involved in mindfulness practices.

Lastly, the rationale for this review was that the development of greater mindfulness capacity might positively impact on cognitive function, due to the theorized relationship between the components of mindfulness and specific neurocognitive systems (Vago & Silbersweig, 2012). However, evaluating whether improvements to cognition do indeed manifest in parallel with increased mindfulness capacity (and whether this relationship holds for specific cognitive domains and mindfulness components) was beyond the scope of this review. Similarly, cognitive effects might be mediated by changes in emotional processing, or vice versa (Vago et al., 2019); these putative mechanisms were not explored by this synthesis. Future investigations are encouraged to delineate these interesting and complex relationships.

## Conclusion

This is the first systematic review and meta-analysis to evaluate objective, cross-domain cognitive outcomes reported by randomized MBP studies. In the primary analysis (pooling data across domains) we identified a small but significant effect on cognitive function. Specific effects were observed for executive function and for working memory (here conceptualized as a subdomain of executive function), but not other individual domains. Small, significant effects were observed for studies of non-clinical, as well as older adult (≥ 60 years) samples, but not for studies of clinical, or adult (< 60 years) samples. In addition, we found that MBPs outperformed inactive, but not active comparators. MBPs place strong emphasis on strengthening mental skills, and this review suggests that this translates to improved performance on objective cognition. Future research is encouraged to adopt more rigorous methodology; to prioritize the standardized measurement and reporting of cognitive function; and to seek to identify the components of MBPs responsible for driving cognitive changes.

## Supplementary Information

Below is the link to the electronic supplementary material.

Supplementary file1 (DOCX 102 KB)

## Data Availability

The datasets used for the present analyses are available via the Open Science Framework: https://osf.io/xru9h/.

## Code Availability

The *R* script necessary to reproduce the present analyses is available via the Open Science Framework: https://osf.io/xru9h/.
