# Supplementary material for: The Effect of Mindfulness-based Programs on Cognitive Function in Adults: A Systematic Review and Meta-analysis
Source: Neuropsychol Rev. 2021 Aug 4;32(3):677–702. doi: 10.1007/s11065-021-09519-y (PMC9381612; doi:10.1007/s11065-021-09519-y)
Supplement: Supplementary file 1 — Supplementary file1 (DOCX 102 KB) [file 11065_2021_9519_MOESM1_ESM.docx]

Supplementary materials

# Manuscript title

The effect of mindfulness-based programs on cognitive function in adults: a systematic review and meta-analysis

# Author list

Tim Whitfield^#^, Thorsten Barnhofer, Rebecca Acabchuk, Avi Cohen, Michael Lee, Marco Schlosser, Eider M. Arenaza-Urquijo, Adriana Böttcher, Willoughby Britton, Nina Coll-Padros, Fabienne Collette, Gaël Chételat, Sophie Dautricourt, Harriet Demnitz-King, Travis Dumais, Olga Klimecki, Dix Meiberth, Inès Moulinet, Theresa Müller, Elizabeth Parsons, Lauren Sager, Lena Sannemann, Jodi Scharf, Ann-Katrin Schild, Edelweiss Touron, Miranka Wirth, Zuzana Walker, Ethan Moitra, Antoine Lutz, Sara W. Lazar, David Vago, Natalie L. Marchant

^#^Corresponding author. Email: [tim.whitfield@ucl.ac.uk](mailto:tim.whitfield@ucl.ac.uk). Telephone: Tel: +44 (0)20 7679 2000. Address: Division of Psychiatry, University College London, Sixth floor Maple House, 149 Tottenham Court Road, London, W1T 7NF, United Kingdom.

# Supplementary Method

## Search strategy: specific search terms used for each resource

### **Academic database literature searches**

#### AMED (Ovid)

| 1. mindful*.ti,ab. |  |
| --- | --- |
| 2. orientation.ti,ab. |  |
| 3. attention*.ti,ab. |  |
| 4. "processing speed".ti,ab. |  |
| 5. percept*.ti,ab. |  |
| 6. memory.ti,ab. |  |
| 7. learn*.ti,ab. |  |
| 8. forget*.ti,ab. |  |
| 9. verbal.ti,ab. |  |
| 10. language.ti,ab. |  |
| 11. construction.ti,ab. |  |
| 12. motor.ti,ab. |  |
| 13. assembl*.ti,ab. |  |
| 14. drawing.ti,ab. |  |
| 15. "concept formation".ti,ab. |  |
| 16. reasoning.ti,ab. |  |
| 17. math*.ti,ab. |  |
| 18. executive.ti,ab. |  |
| 19. cogniti*.ti,ab. |  |
| 20. neuropsycholog*.ti,ab. |  |
| 21. 2 or 3 or 4 or 5 or 6 or 7 or 8 or 9 or 10 or 11 or 12 or 13 or 14 or 15 or 16 or 17 or 18 or 19 or 20 |  |
| 22. evaluat*.ti,ab,sh. |  |
| 23. effective*.ti,ab,sh. |  |
| 24. parallel.ti,ab,sh. |  |
| 25. trial*.ti,ab,sh. |  |
| 26. random*.ti,ab,sh. |  |
| 27. RCT.ti,ab,sh. |  |
| 28. "interventional stud*".ti,ab,sh. |  |
| 29. "intervention stud*".ti,ab,sh. |  |
| 30. "controlled stud*".ti,ab,sh. |  |
| 31. "experimental stud*".ti,ab,sh. |  |
| 32. "comparative stud*".ti,ab,sh. |  |
| 33. "prospective stud*".ti,ab,sh. |  |
| 34. pilot*.ti,ab,sh. |  |
| 35. 22 or 23 or 24 or 25 or 26 or 27 or 28 or 29 or 30 or 31 or 32 or 33 or 34 |  |
| 36. 1 and 21 and 35 |  |

#### CINAHL Plus

| S1 | TI mindful* OR AB mindful* |
| --- | --- |
| S2 | (MH "Randomized Controlled Trials") OR (MH "Clinical Trials") OR (MH "Preventive Trials") OR (MH "Intervention Trials") OR (MH "Double-Blind Studies") OR (MH "Single-Blind Studies") OR (MH "Therapeutic Trials") OR (MH "Triple-Blind Studies") |
| S3 | TI evaluat* OR effective* OR pilot* OR parallel OR trial* OR random* OR RCT OR “interventional stud*” OR “intervention stud*” OR “experimental stud*” OR “controlled stud*” OR “comparative stud*” OR “prospective stud*” |
| S4 | AB evaluat* OR effective* OR pilot* OR parallel OR trial* OR random* OR RCT OR “interventional stud*” OR “intervention stud*” OR “experimental stud*” OR “controlled stud*” OR “comparative stud*” OR “prospective stud*” |
| S5 | SU evaluat* OR effective* OR pilot* OR parallel OR trial* OR random* OR RCT OR “interventional stud*” OR “intervention stud*” OR “experimental stud*” OR “controlled stud*” OR “comparative stud*” OR “prospective stud*” |
| S6 | TI ( orientation OR attention* OR “processing speed” OR percept* OR memory OR learn* OR forget* OR math* OR verbal OR language OR construction OR assembl* or drawing OR motor OR "concept formation" OR reasoning OR “executive function*” OR cogniti* OR neuropsycholog* ) OR AB ( orientation OR attention* OR “processing speed” OR percept* OR memory OR learn* OR forget* OR math* OR verbal OR language OR construction OR assembl* or drawing OR motor OR "concept formation" OR reasoning OR “executive fun [...](javascript:showHistoryTerm('ctl00_ctl00_MainContentArea_MainContentArea_historyControl_HistoryRepeater_ctl02_ellipsis',true)) |
| S7 | S2 OR S3 OR S4 OR S5 |
| S8 | S1 AND S6 AND S7 |

#### EMBASE (Ovid)

| 1. mindful*.ti,ab. |  |
| --- | --- |
| 2. orientation.ti,ab. |  |
| 3. attention*.ti,ab. |  |
| 4. "processing speed".ti,ab. |  |
| 5. percept*.ti,ab. |  |
| 6. memory.ti,ab. |  |
| 7. learn*.ti,ab. |  |
| 8. forget*.ti,ab. |  |
| 9. verbal.ti,ab. |  |
| 10. language.ti,ab. |  |
| 11. construction.ti,ab. |  |
| 12. motor.ti,ab. |  |
| 13. assembl*.ti,ab. |  |
| 14. drawing.ti,ab. |  |
| 15. "concept formation".ti,ab. |  |
| 16. reasoning.ti,ab. |  |
| 17. math*.ti,ab. |  |
| 18. executive.ti,ab. |  |
| 19. cogniti*.ti,ab. |  |
| 20. neuropsycholog*.ti,ab. |  |
| 21. 2 or 3 or 4 or 5 or 6 or 7 or 8 or 9 or 10 or 11 or 12 or 13 or 14 or 15 or 16 or 17 or 18 or 19 or 20 |  |
| 22. evaluat*.ti,ab,sh. |  |
| 23. effective*.ti,ab,sh. |  |
| 24. parallel.ti,ab,sh. |  |
| 25. trial*.ti,ab,sh. |  |
| 26. random*.ti,ab,sh. |  |
| 27. RCT.ti,ab,sh. |  |
| 28. "interventional stud*".ti,ab,sh. |  |
| 29. "intervention stud*".ti,ab,sh. |  |
| 30. "controlled stud*".ti,ab,sh. |  |
| 31. "experimental stud*".ti,ab,sh. |  |
| 32. "comparative stud*".ti,ab,sh. |  |
| 33. "prospective stud*".ti,ab,sh. |  |
| 34. pilot*.ti,ab,sh. |  |
| 35. exp Experimental Design/ |  |
| 36. exp intervention study/ |  |
| 37. exp "phase 2 clinical trial (topic)"/ or exp superiority trial/ or exp equivalence trial/ or exp "phase 3 clinical trial (topic)"/ or exp pragmatic trial/ or exp "controlled clinical trial (topic)"/ or exp "phase 4 clinical trial (topic)"/ or exp controlled clinical trial/ or exp non-inferiority trial/ or exp "clinical trial (topic)"/ or exp "randomized controlled trial (topic)"/ or exp "phase 1 clinical trial (topic)"/ |  |
| 38. 22 or 23 or 24 or 25 or 26 or 27 or 28 or 29 or 30 or 31 or 32 or 33 or 34 or 35 or 36 or 37 |  |
| 39. 1 and 21 and 38 |  |

#### MEDLINE (Ovid)

| 1. mindful*.ti,ab. |  |
| --- | --- |
| 2. orientation.ti,ab. |  |
| 3. attention*.ti,ab. |  |
| 4. "processing speed".ti,ab. |  |
| 5. percept*.ti,ab. |  |
| 6. memory.ti,ab. |  |
| 7. learn*.ti,ab. |  |
| 8. forget*.ti,ab. |  |
| 9. verbal.ti,ab. |  |
| 10. language.ti,ab. |  |
| 11. construction.ti,ab. |  |
| 12. motor.ti,ab. |  |
| 13. assembl*.ti,ab. |  |
| 14. drawing.ti,ab. |  |
| 15. "concept formation".ti,ab. |  |
| 16. reasoning.ti,ab. |  |
| 17. math*.ti,ab. |  |
| 18. executive.ti,ab. |  |
| 19. cogniti*.ti,ab. |  |
| 20. neuropsycholog*.ti,ab. |  |
| 21. 2 or 3 or 4 or 5 or 6 or 7 or 8 or 9 or 10 or 11 or 12 or 13 or 14 or 15 or 16 or 17 or 18 or 19 or 20 |  |
| 22. evaluat*.ti,ab,sh. |  |
| 23. effective*.ti,ab,sh. |  |
| 24. parallel.ti,ab,sh. |  |
| 25. trial*.ti,ab,sh. |  |
| 26. random*.ti,ab,sh. |  |
| 27. RCT.ti,ab,sh. |  |
| 28. "interventional stud*".ti,ab,sh. |  |
| 29. "intervention stud*".ti,ab,sh. |  |
| 30. "controlled stud*".ti,ab,sh. |  |
| 31. "experimental stud*".ti,ab,sh. |  |
| 32. "comparative stud*".ti,ab,sh. |  |
| 33. "prospective stud*".ti,ab,sh. |  |
| 34. pilot*.ti,ab,sh. |  |
| 35. exp RANDOMIZED CONTROLLED TRIAL/ |  |
| 36. exp CLINICAL TRIAL/ |  |
| 37. 22 or 23 or 24 or 25 or 26 or 27 or 28 or 29 or 30 or 31 or 32 or 33 or 34 or 35 or 36 |  |
| 38. 1 and 21 and 37 |  |

#### PsycINFO/PsycBOOKS (Ovid)

| 1. mindful*.ti,ab. |  |
| --- | --- |
| 2. orientation.ti,ab. |  |
| 3. attention*.ti,ab. |  |
| 4. "processing speed".ti,ab. |  |
| 5. percept*.ti,ab. |  |
| 6. memory.ti,ab. |  |
| 7. learn*.ti,ab. |  |
| 8. forget*.ti,ab. |  |
| 9. verbal.ti,ab. |  |
| 10. language.ti,ab. |  |
| 11. construction.ti,ab. |  |
| 12. motor.ti,ab. |  |
| 13. assembl*.ti,ab. |  |
| 14. drawing.ti,ab. |  |
| 15. "concept formation".ti,ab. |  |
| 16. reasoning.ti,ab. |  |
| 17. math*.ti,ab. |  |
| 18. executive.ti,ab. |  |
| 19. cogniti*.ti,ab. |  |
| 20. neuropsycholog*.ti,ab. |  |
| 21. 2 or 3 or 4 or 5 or 6 or 7 or 8 or 9 or 10 or 11 or 12 or 13 or 14 or 15 or 16 or 17 or 18 or 19 or 20 |  |
| 22. evaluat*.ti,ab,sh. |  |
| 23. effective*.ti,ab,sh. |  |
| 24. parallel.ti,ab,sh. |  |
| 25. trial*.ti,ab,sh. |  |
| 26. random*.ti,ab,sh. |  |
| 27. RCT.ti,ab,sh. |  |
| 28. "interventional stud*".ti,ab,sh. |  |
| 29. "intervention stud*".ti,ab,sh. |  |
| 30. "controlled stud*".ti,ab,sh. |  |
| 31. "experimental stud*".ti,ab,sh. |  |
| 32. "comparative stud*".ti,ab,sh. |  |
| 33. "prospective stud*".ti,ab,sh. |  |
| 34. pilot*.ti,ab,sh. |  |
| 35. exp INTERVENTION/ |  |
| 36. exp Experimental Design/ |  |
| 37. 22 or 23 or 24 or 25 or 26 or 27 or 28 or 29 or 30 or 31 or 32 or 33 or 34 or 35 or 36 |  |
| 38. 1 and 21 and 37 |  |

#### SCOPUS

( TITLE-ABS ( mindful* ) AND TITLE-ABS-KEY ( evaluat* OR effective* OR parallel OR trial* OR random* OR rct OR "interventional stud*" OR "intervention stud*" OR "controlled stud*" OR "experimental stud*" OR "comparative stud*" OR "prospective stud*" OR pilot* ) AND TITLE-ABS ( orientation OR attention* OR "processing speed" OR percept* OR memory OR learn* OR forget* OR verbal OR language OR construction OR motor OR assembl* OR drawing OR "concept formation" OR reasoning OR math* OR executive OR cogniti* OR neuropsycholog* ) )

#### Web of Science

TOPIC: (mindful* and (orientation or attention* or "processing speed" or percept* or memory or learn* or forget* or verbal or language or construction or motor or assembl* or drawing or "concept formation" or reasoning or math* or executive or cogniti* or neuropsycholog*) and (evaluat* OR effective* OR parallel OR trial* OR random* OR RCT OR “interventional stud*” OR “intervention stud*” OR “controlled stud*” OR “experimental stud*” OR “comparative stud*” OR “prospective stud*” OR pilot*))

Indexes=SCI-EXPANDED, SSCI, CPCI-S, CPCI-SSH, BKCI-S, BKCI-SSH Timespan=All years

### **Grey literature searches**

#### OpenGrey

mindful*

#### ProQuest Dissertations & Theses Global

(ti(mindful*) OR ab(mindful*)) AND (su(approach* OR "comparative stud*" OR "controlled stud*" OR "experimental stud*" OR intervention* OR program* OR random* OR RCT OR therap* OR training OR treatment OR trial*) OR ti(approach* OR "comparative stud*" OR "controlled stud*" OR "experimental stud*" OR intervention* OR program* OR random* OR RCT OR therap* OR training OR treatment OR trial*) OR ab(approach* OR "comparative stud*" OR "controlled stud*" OR "experimental stud*" OR intervention* OR program* OR random* OR RCT OR therap* OR training OR treatment OR trial*)) AND (ti(orientation OR attention* OR "processing speed" OR percept* OR memory OR learn* OR forget* OR math* OR verbal OR language OR construction OR assembl* OR drawing OR motor OR "concept formation" OR reasoning OR "executive function*" OR cogniti* OR neuropsycholog*) OR ab(orientation OR attention* OR "processing speed" OR percept* OR memory OR learn* OR forget* OR math* OR verbal OR language OR construction OR assembl* OR drawing OR motor OR "concept formation" OR reasoning OR "executive function*" OR cogniti* OR neuropsycholog*))

#### Clinicaltrials.gov

**Advanced search ‘other terms’**

(mindfulness OR mindful) AND (orientation OR attention OR attentional OR "processing speed" OR perception OR perceptual OR memory OR learn OR learning OR learned OR learnt OR forget OR forgetting OR verbal OR language OR math OR maths OR mathematic OR mathematical OR verbal OR language OR construction OR assemble OR assembling OR assembly OR drawing OR motor OR "concept formation" OR reasoning OR executive OR cognitive OR cognition OR neuropsychology OR neuropsychological)

#### Google Scholar

**(Only the first 300 hits were accessed)**

mindfulness AND (attention OR "processing speed" OR perception OR memory OR learning OR verbal OR language OR motor OR "concept formation" OR reasoning OR "executive function" OR cognition OR cognitive OR neuropsychology OR neuropsychological)

# Supplementary Method

# Statistical analyses

## Calculation of effect sizes

# The measure of effect size was the standardized mean difference, with approximate correction factor *J* (referred to as *c_P_* in the formula below) applied for small sample sizes. In the main paper, the effect size *g* (as well as its variance) were calculated as per the formula presented for *d_ppc2_* (here, ‘ppc’ refers to the intended use of this effect size measure in pretest-posttest-control studies) in Morris (2007). In the following series of equations, the following nomenclature is used:

| **Operator** | **Definition** |
| --- | --- |
| *M* | Mean score on a cognitive test outcome |
| *_T_* | Treatment group |
| *_C_* | Comparator group |
| *_Post_* | Immediately post-intervention |
| *_Pre_* | Prior to intervention (i.e. baseline) |
| *n* | Number of participants in a group |
| *ρ* | Correlation between M_pre_ and M_post_ |
| *c_P_* | Approximate common small sample bias adjustment |
| *SD* | Standard deviation |
| △ | Effect size |

The effect size formula for *d_ppc2_* follows equation (8) in Morris (2007):

$$d_{ppc2}=c_{P} \left[ \frac{\left( M_{post,T}-M_{pre,T} \right)-(M_{post,C}-M_{pre,C})}{{SD}_{pre}} \right]$$

Where the pooled standard deviation is defined as equation (9):

$${SD}_{pre}= \sqrt{\frac{\left( n_{T}-1 \right){SD}_{pre,T}^{2}+(n_{C}-1){SD}_{pre,C}^{2}}{n_{T}-n_{C}-2}}$$

And the small sample bias adjustment is defined according to equation (10):

$$c_{P}=1-\frac{3}{4\left( n_{T}+ n_{C}-2 \right)-1}$$

The variance of *d_ppc2_* is given by equation (25):

$$\sigma^{2}\left( d_{ppc2} \right)=2\left( c_{P}^{2} \right)\left( 1-\rho\right)\left( \frac{n_{T}+ n_{C}}{n_{T} n_{C}} \right)\left( \frac{n_{T}+ n_{C}-2}{n_{T}+ n_{C}-4} \right)\left( 1+\frac{\triangle^{2}}{2\left( 1-\rho\right)\left( \frac{n_{T}+ n_{C}}{n_{T} n_{C}} \right)} \right)-\triangle^{2}$$

Note, based on convention and empirical observations, *ρ* (correlation between M_pre_ and M_post_) was fixed at 0.5 throughout all analyses in the review. In this case:

$$2\left( 1-\rho\right)=1$$

and is thus effectively omitted from the employed formula (given its exclusive use as a multiplier of other terms).

# Supplementary Results

## Follow-up

Whilst there were not enough studies to meta-analyze effects at follow-up, available results are briefly described here. Of the seven studies utilizing short-term (8–18 week) follow-ups, one identified an effect on executive function favoring the MBP at follow-up (Johns et al., 2016). Two studies reported mixed evidence, depending on the statistical analyses, on attention and executive function, respectively (MacCoon et al., 2014; Webb et al., 2018). One study reported no difference between the MBP and control group at follow-up (various cognitive domains; Larouche et al. (2018)), and the remaining three studies did not report cognitive outcomes at follow-up. Of the five studies utilizing long-term (24–44 week) follow-ups, one found a trend for executive function improvement (Lebares et al., 2019), two found no difference between the MBP and control arms on executive function (Jermann et al., 2013; Moynihan et al., 2013), and the remaining two studies did not report cognitive data at follow-up. There was thus a lack of data on which to base evaluation of effects at follow-up, although where available, effects were mixed. More data will be required to inform more confident conclusions in future.

## Cognitive domains (visual perception and construction)

Two actively-controlled studies included measures of visual perception (Baird et al., 2014; Jensen et al., 2012); neither reported significant time by group interactions. Five studies (three actively- and two inactively-controlled) administered tests from the construction domain (Bubb, 2014; Fam et al., 2020; Ives-Deliperi et al., 2013; Tang et al., 2015; Wetherell et al., 2017). No study reported significant time by group interactions on these measures.

# Supplementary Tables and Figures

**Table S1**

Summary of sample demographic and intervention characteristics from the systematic review (*k* = 56)

| **Factor** | **Median (range) or Percent** |
| --- | --- |
| Location |  |
| *North America* | 43% |
| *Europe* | 32% |
| *Asia* | 16% |
| *Other^#^* | 9% |
| Sample size | 43 (14 to 200) |
| Female | 67% |
| Age (years) | 41 (19 to 82) |
| Age groups |  |
| *Adults (mean age < 60 years)* | 71% |
| *Older adults (mean age* ≥ *60 years)* | 29% |
| Clinical status |  |
| *Non-clinical* | 61% |
| *Clinical* | 39% |
| *Neurocognitive disorders* | 14% |
| *Psychiatric disorders* | 14% |
| *Neurological disorders* | 7% |
| *Cancer* | 2% |
| *HIV* | 2% |
| Ethnicity |  |
| *White* | 71% |
| *Asian* | 15% |
| *Black* | 9% |
| *Other, or data unavailable* | 5% |
| Type of MBP |  |
| *Modified* | 54% |
| *Generic* | 23% |
| *Unmodified MBSR* | 18% |
| *Unmodified MBCT* | 5% |
| Primary comparator type |  |
| *Active* | 46% |
| *Waitlist* | 36% |
| *Treatment as Usual* | 11% |
| *No intervention* | 7% |

For some categories, the given figures only reflect studies with available data – please see main paper for the number of studies contributing data to each row. **Abbreviations** – HIV = Human Immunodeficiency Virus; MBP = Mindfulness-based program; MBSR = Mindfulness-Based Stress Reduction; MBCT = Mindfulness-Based Cognitive Therapy. **Superscript** – ^#^Comprising two studies in Australia, two in Israel and one in South Africa.

**Table S2**

Comparing sample demographic characteristics between the MBP arm and main comparator

| **Characteristic** | ***K*** | ***N***  (MBP / Comparator) | **MBP** | **Comparator** |
| --- | --- | --- | --- | --- |
| Age (x̅, years) | 43 | 1,222 / 1,180 | 49.3 | 49.4 |
| Sex (female, %) | 45 | 1,279 / 1,249 | 65.7 | 67.0 |
| Education (x̅, years) | 12 | 399 / 406 | 14.9 | 14.7 |
| Ethnicity (%) | 16 | 370 / 368 |  |  |
| White |  |  | 58.1 | 61.1 |
| Asian |  |  | 22.7 | 21.5 |
| Black |  |  | 15.1 | 12.2 |
| Other/Unknown |  |  | 4.1 | 5.2 |

Some studies only reported the sample grand mean or proportion for the above characteristics (i.e. data were not broken down by arm). Thus, the numbers of studies contributing data to the comparisons above were smaller than the numbers informing the paragraph ‘Participant characteristics’ in the main paper’s Results. **Abbreviations** – *K* = Number of studies; MBP = Mindfulness-based program**.**

**Table S3**

Specific measures included in the main meta-analysis, organized by cognitive domain/subdomain

| **Measure name** | **Subscale/sub-score** (and calculation details where applicable) | **Measurement unit** | ***K*** |
| --- | --- | --- | --- |
| **EXECUTIVE FUNCTION – Cognitive flexibility** | | | |
| Barcelona Test-Revised: Category Fluency | Total - Correct | Items | 1 |
|  | Total - Perseverations | Items | 1 |
| Barcelona Test-Revised: Letter Fluency | Total - Correct | Items | 1 |
|  | Total - Perseverations | Items | 1 |
| BRB-N: Word List Generation | Total - Correct | Items | 1 |
| Category Fluency | Total - Correct | Items | 1 |
| Letter Fluency | Total - Correct | Items | 2 |
| NIH EXAMINER | Cognitive control index | *NC* | 1 |
|  | Verbal fluency index | *NC* | 1 |
| Plus-Minus Task | Shifting cost - Time | Percentage | 1 |
| Stroop | Switching | Seconds | 1 |
| Switching Task | Switch cost (between switch and non-switch blocks) | Milliseconds (RT) | 1 |
|  | Switch cost (between switch and non-switch trials) | Milliseconds (RT) | 1 |
| Trail Making Test | Trails ratio (B/A) - Time | Seconds | 1 |
|  | Trails cost (B-A) - Errors | Items | 1 |
|  | Trails cost (B-A) - Time | Seconds | 1 |
| Wisconsin Card Sorting Test | Number of categories completed | Items | 1 |
|  | Perseverative errors | Percentage | 1 |
| **EXECUTIVE FUNCTION – Working memory** | | | |
| Continuous Performance Test (AX-CPT) | Behavior shift index - Error rate ((AY – BX)/(AY + BX)) | *NC* | 1 |
|  | Behavior shift index - Reaction time ((AY – BX)/(AY + BX)) | Milliseconds (RT) | 1 |
| Digit Span | Mixed | Items | 1 |
|  | *NA* | Items | 1 |
|  | Backwards | Items | 5 |
| NIH EXAMINER | Working memory index | *NC* | 1 |
| Operation Span | Math paradigm - Errors | Items | 1 |
|  | Total - Correct | Items | 1 |
|  | Total - Correct | Percentage | 1 |
|  | Stringent score - Correct | Items | 1 |
| The Dual-Back Task (2-Back) | *NA* | *NC* | 1 |
| WAIS-III: Arithimetic | *NA* | *NC* | 1 |
| WAIS-III: Direct Digits | *NA* | *NC* | 1 |
| WAIS-III: Inverse Digits | *NA* | *NC* | 1 |
| WAIS-IV: Digit Span | Sequencing | Items | 1 |
| WAIS-IV: Letter-Number Sequencing | *NA* | Items | 1 |
| Word version of the Continuous Performance Task | Proactivity index | SDT-derived sensitivity measure | 1 |
| **EXECUTIVE FUNCTION – Inhibition** | | | |
| Attention Network Test | Executive control network effect - Reaction time | Milliseconds (RT) | 6 |
|  | Executive control network effect - Errors | Items | 1 |
| Auditory Go/No-Go Task | Post-error slowdown | Milliseconds (RT) | 1 |
| Computerised Stroop | Interference (incongruent minus congruent) | Milliseconds (RT) | 1 |
|  | Interference (incongruent minus neutral) | Milliseconds (RT) | 1 |
| Flanker Task | Conflict score (incongruent minus congruent) | Milliseconds (RT) | 1 |
| Reading-with-Distraction Task | *NA* | Seconds | 1 |
| Six Letter Cancellation Test | Commission errors | Items | 1 |
| Stroop | *NA* | *NC* | 1 |
|  | Inhibition | Seconds | 1 |
|  | Interference (incongruent minus congruent) - Accuracy | Percentage | 1 |
|  | Interference (incongruent minus congruent) - Errors | Items | 1 |
|  | Interference (incongruent minus congruent) - Time | Seconds | 2 |
| Stroop Color and Word Test – Victoria version | Interference (incongruent minus neutral) - Time | Seconds | 1 |
| Sustained Attention to Response Task | Post-error slowdown | Milliseconds (RT) | 1 |
| Theory of Visual Attention Test | Attentional selectivity | *NC* | 1 |
| Word version of the Continuous Performance Task | Reactivity index | SDT-derived sensitivity measure | 1 |
| **ATTENTION – Alerting** | | | |
| Attention Network Test | Alerting network effect - Reaction time | Milliseconds (RT) | 6 |
|  | Alerting network effect - Errors | Items | 1 |
| Auditory Oddball Task | Coefficient of variation - Reaction time | Milliseconds (RT) | 1 |
| BRB-N: Paced Auditory Serial Addition Test | 2-second condition - Correct | Items | 1 |
|  | 3-second condition - Correct | Items | 1 |
| BRB-N: Symbol Digit Modalities Test | Total - Correct | Items | 1 |
| CANTAB: RVP | *NA* | SDT-derived sensitivity measure | 1 |
| Continuous Performance Test | Sensitivity index | SDT-derived sensitivity measure | 1 |
|  | Variability - Reaction time | Milliseconds (RT) | 1 |
|  | Variability | *NC* | 1 |
|  | Detectability | *NC* | 1 |
| D2 Test of Attention | Hits minus errors of commission | Items | 1 |
|  | Total number of responses minus omissions plus commissions | Items | 1 |
|  | Total responses minus errors | Items | 1 |
| Digit Symbol Coding | *NA* | Items | 2 |
| Dual Attention to Response Task | Coefficient of variation - Reaction time | Milliseconds (RT) | 1 |
| Go/No-Go | Sensitivity index | SDT-derived sensitivity measure | 1 |
|  | Coefficient of variation - Reaction time | Milliseconds (RT) | 1 |
| Letter-Digit Substitution Test | *NA* | Items | 1 |
| Multiple Objects Tracking Task | Individual trial - Accuracy | Percentage | 1 |
| Six Letter Cancellation Test | Completion - Time | Seconds | 1 |
|  | Omission errors | Items | 1 |
| Spatial & Temporal Attention Network Task | Neutral | Milliseconds (RT) | 1 |
|  | Temporal invalid | Milliseconds (RT) | 1 |
| Sustained Attention to Response Task | Accuracy index | SDT-derived sensitivity measure | 1 |
|  | Intra-individual coefficient of variation | *NC* | 1 |
|  | Coefficient of variation - Reaction time | Milliseconds (RT) | 1 |
| Verbal Cancellation | Total - Errors | Items | 1 |
|  | Total - Time | Seconds | 1 |
| Vigil Continuous Performance Test | Individual trial hit rate minus false alarm rate | Items | 1 |
| Visual Cancellation | Total - Errors | Items | 1 |
|  | Total - Time | Seconds | 1 |
| Wilkins Counting Task | Error rate | Percentage | 1 |
| Word version of the Continuous Performance Task | Coefficient of variation - Reaction time | Milliseconds (RT) | 1 |
| **ATTENTION – Orienting** | | | |
| Attention Network Test | Orienting network effect - Errors | Items | 1 |
|  | Orienting network effect - Reaction time | Milliseconds (RT) | 5 |
| **DECLARATIVE MEMORY – Episodic memory** | | | |
| ADAS-COG: Word List Learning | Delayed - Correct | Items | 1 |
|  | Immediate - Correct | Items | 1 |
| BRB-N: 10/36 Spatial Recall Test | Delayed - Correct | Items | 1 |
|  | Immediate - Correct | Items | 1 |
| BRB-N: Selective Reminding Test | Consistent long-term retrieval - Correct | Items | 1 |
|  | Delayed - Correct | Items | 1 |
|  | Long-term storage - Correct | Items | 1 |
| Chinese Auditory Verbal Learning Test | Delayed recall - Correct | Items | 1 |
|  | Delayed recognition - Correct | Items | 1 |
|  | Delayed recognition - Errors | Items | 1 |
|  | Intrusion errors | Items | 1 |
|  | Recall after interference - Correct | Items | 1 |
|  | Trials 1 to 5 - Sum correct | Items | 1 |
| Episodic Memory Task | Consolidation | Percentage | 1 |
|  | Delayed recall - Correct | Items | 1 |
|  | Recognition - Correct | Items | 1 |
|  | Gains | Percentage | 1 |
|  | Immediate recall - Correct | Items | 1 |
|  | Losses | Percentage | 1 |
| Memory Recognition Task | Hits minus false alarms | Items | 1 |
| Rey Auditory Verbal Learning Test | Delayed recall - Correct | Items | 3 |
|  | Delayed recall - Intrusions | Items | 1 |
|  | Delayed recall - Perseverations | Items | 1 |
|  | Recognition - Correct | Items | 2 |
|  | Recognition - Errors | Items | 1 |
|  | Immediate - Correct | Items | 1 |
|  | Trial 5 minus trial 6 - Correct | Items | 1 |
|  | Trial 6 - Correct | Items | 1 |
|  | Trial 6 - Intrusions | Items | 1 |
|  | Trial 6 - Perseverations | Items | 1 |
|  | Trials 1 to 5 - Sum Correct | Items | 1 |
|  | Trials 1 to 5 - Sum Intrusions | Items | 1 |
|  | Trials 1 to 5 - Sum Perseverations | Items | 1 |
| Rey Complex Figure Test | Delayed recall - Correct | *NC* | 1 |
|  | Delayed recognition - Correct | *NC* | 1 |
|  | Immediate recall - Correct | *NC* | 1 |
| Visual Memory | Total - Correct | *NC* | 1 |
| WMS-R: Logical Memory Test | Immediate - Correct | Items | 1 |
| **DECLARATIVE MEMORY – Short-term memory** | | | |
| Digit Span | Forwards | Items | 5 |
| One-Back Letter Task | Correct responses | Percentage | 1 |
|  | Reaction time | Milliseconds (RT) | 1 |
| Theory of Visual Attention Test | Capacity of visual short-term memory | Items | 1 |
| **COGNITIVE AGING** |  |  |  |
| ADAS-COG | Total - Errors | Items | 2 |
| Mini-Mental State Examination | Total - Correct | Items | 5 |
| **CONSTRUCTION** |  |  |  |
| Grooved Pegboard | Total - Time | Seconds | 1 |
| Hooper Visual Organization Test | *NA* | *NC* | 1 |
| Rey Complex Figure | Copy | Seconds | 1 |
| **VISUAL PERCEPTION** | | | |
| WAIS-IV: Block Design | *NA* | Seconds | 2 |
| Perceptual Threshold | *NA* | Degrees | 1 |
| Theory of Visual Attention Test | Perceptual threshold | *NC* | 1 |
|  | Processing speed | *NC* | 1 |

This table describes all the cognitive outcome measures contributing effect sizes to the final meta-analysis (*k* = 45; *N* effect sizes = 179). **Abbreviations** – *K* = Number of studies (frequency); BRB-N = Brief Repeatable Battery of Neuropsychological Tests for Multiple Sclerosis; NIH EXAMINER = National Institutes for Health-Executive Abilities: Measures and Instruments for Neurobehavioral Evaluation and Research; WAIS = Wechsler Adult Intelligence Scale; CANTAB: RVP = Cambridge Neuropsychological Test Automated Battery: Rapid Visual Processing; ADAS-COG = Alzheimer’s Disease Assessment Scale – Cognitive Subscale; WMS-R = Wechsler Memory Scale—Revised; RT = Reaction time; SDT = Signal detection theory; *NA* = Not applicable; *NC* = Not clear.

**Table S4**

Univariable meta-regression of standard error on effect size to evaluate potential publication bias

| **Subgroup** | ***K* (*N* ES)** | **Estimate** | **Estimate** | **95% CI** | ***df*** | ***p*-value** |
| --- | --- | --- | --- | --- | --- | --- |
| All studies | 45 (179) | Intercept | 0.33 | [-0.01, 0.68] | 10 | .058 |
|  |  | Effect size *SE* | -0.63 | [-1.64, 0.38] | 13 | .200 |
| Actively-controlled | 22 (84) | Intercept | 0.10 | [-0.51, 0.72] | 6 | .700 |
|  |  | Effect size *SE* | -0.10 | [-1.97, 1.78] | 7 | .906 |
| Inactively-controlled* | 28 (120) | Intercept | **0.46** | [0.10, 0.82] | 6 | .021 |
|  |  | Effect size *SE* | -0.83 | [-1.82, 0.15] | 9 | .087 |

Effects in **bold** reached statistical significance (*p* < .05). The heterogeneity statistics for these models are as follows: All studies, I^2^ = 18.54 and Tau^2^ = 0.02; Actively-controlled studies, I^2^ = 0.00 and Tau^2^ = 0.00; Inactively-controlled studies, I^2^ = 24.25 and Tau^2^ = 0.03. **Abbreviations** – *K* = Number of studies; ES = Effect size; CI = Confidence interval; *df* = Degrees of freedom; *SE* = Standard error. **Superscript** – *Includes an additional inactive arm from each of the five studies which featured both active and inactive comparators.

**Table S5**

Multivariable meta-regression

| **Parameter** | **Estimate** | **95% CI** | ***df*** | ***p*-value** |
| --- | --- | --- | --- | --- |
| Intercept | 0.20 | [-0.69, 1.09] | 11 | 0.631 |
| Inactively-controlled^±^ | 0.20 | [-0.16, 0.57] | 14 | 0.254 |
| Older adults (≥ 60 years)^$^ | 0.05 | [-0.28, 0.38] | 13 | 0.735 |
| Non-clinical^#^ | -0.00 | [-0.26, 0.25] | 20 | 0.973 |
| MBP type |  |  |  |  |
| Unmodified MBSR/MBCT^£^ | -0.10 | [-0.80, 0.61] | 10 | 0.262 |
| Modified MBP^£^ | -0.02 | [-0.51, 0.47] | 9 | 0.321 |
| Number of formal mindfulness practices | -0.04 | [-0.35, 0.27] | 11 | 0.787 |
| MBP retreat included* | 0.06 | [-0.31, 0.43] | 9 | 0.713 |
| Number of MBP sessions | -0.02 | [-0.05, 0.02] | 8 | 0.276 |
| Frequency of MBP sessions | 0.07 | [-0.22, 0.36] | 11 | 0.581 |
| Duration of MBP sessions (mins)^€^ | -0.00 | [-0.01, 0.00] | 11 | 0.550 |

This meta-regression analysis was repeated, substituting age in years for age groups, and results were essentially unchanged. Three studies were excluded from analyses – Bowden et al. (2012) did not report the sample age, and a further two studies did not report the MBP session duration (Becerra et al., 2017; Ives-Deliperi et al., 2013). A total of 169 effect sizes from 42 studies were thus included. For each study, only the main comparator was included. The heterogeneity statistics for this model are I^2^ = 22.88 and Tau^2^ = 0.03. **Abbreviations** – MBP = Mindfulness-based program; MBSR = Mindfulness-Based Stress Reduction; MBCT = Mindfulness-Based Cognitive Therapy; CI = Confidence interval; *df* = Degrees of freedom. **Superscripts** – ^±^Versus actively-controlled studies; ^$^Versus adult samples (< 60 years); ^#^Versus clinical samples; ^£^Versus generic MBPs; *Versus studies not including an MBP retreat; ^€^Mean-centered at meta-analytic x̅ (109 minutes).

**Table S6**

Exploratory meta-analyses of Attention Network Test sub-scores

| Network effect | *K* (*N* ES) | *ES (g)* | 95% CI | *df* | *p*-value | Tau^2^ | I^2^ |
| --- | --- | --- | --- | --- | --- | --- | --- |
| Alerting | 6 (7) | -0.15 | [-0.31, 0.01] | 4 | .057 | 0.00 | 0.00 |
| Orienting | 5 (6) | 0.15 | [-0.41, 0.71] | 3* | * | 0.09 | 45.79 |
| Executive | 6 (7) | 0.09 | [-0.24, 0.42] | 4 | .495 | 0.00 | 0.00 |

**Abbreviations** – *K* = Number of studies; ES = Effect size; *g* = Hedges’ standardized mean difference (positive values imply improvement); CI = Confidence interval; *df* = Degrees of freedom. **Superscript** – *Where *df* < 4, *p*-values are unreliable, and are thus not reported here.

**Table S7**

Exploratory meta-analyses for Older adults (≥ 60 years) by separate cognitive domains

| Domain | *K* (*N* ES) | *ES (g)* | 95% CI | *df* | *p*-value | Tau^2^ | I^2^ |
| --- | --- | --- | --- | --- | --- | --- | --- |
| Executive function | 8 (21) | **0.27** | [0.05, 0.50] | 5 | .026 | 0.02 | 19.05 |
| Attention | 7 (17) | 0.12 | [-0.20, 0.44] | 5 | .387 | 0.02 | 20.81 |
| Declarative memory | 6 (16) | 0.15 | [-0.15, 0.46] | 3* | * | 0.01 | 9.75 |

Effects in **bold** reached statistical significance (*p* < .05). Limited data precluded the analysis of cognitive subdomains. The data for the Cognitive aging domain are not included here, as these would be identical to those displayed in Table 2 (see main paper). **Abbreviations** – *K* = Number of studies; ES = Effect size; *g* = Hedges’ standardized mean difference (positive values imply improvement); CI = Confidence interval; *df* = Degrees of freedom. **Superscript** – *Where *df* < 4, *p*-values are unreliable, and are thus not reported here.

**Table S8**

Exploratory meta-analyses of older adult studies, broken down by clinical status

| Subgroup | *K* (*N* ES) | *ES (g)* | 95% *CI* | *df* | *p*-value | Tau^2^ | I^2^ |
| --- | --- | --- | --- | --- | --- | --- | --- |
| Older, Clinical | 7 (22) | 0.11 | [-0.08, 0.29] | 3* | * | 0.00 | 0.00 |
| Older, Non-clinical | 8 (42) | 0.25 | [-0.01, 0.51] | 6 | .057 | 0.03 | 33.87 |

**Abbreviations** – *K* = Number of studies; ES = Effect size; *g* = Hedge’s standardized mean difference (positive values imply improvement); CI = Confidence interval; *df* = Degrees of freedom. **Superscript** – * Where *df* < 4, *p*-values are unreliable, and are thus not reported here.

**Table S9**

Exploratory meta-analyses of MBP type broken down by comparator type

| **MBP type** | **Active comparators** | | **Inactive comparators*** | |
| --- | --- | --- | --- | --- |
|  | ***K* (*N* ES)** | **ES *(g)* [95% CI]** | ***K* (*N* ES)** | **ES *(g)* [95% *CI*]** |
| MBSR/MBCT | 4 (11) | *-0.03* [-0.69, 0.64] | 10 (37) | 0.09 [-0.19, 0.37] |
| Modified MBP | 12 (62) | 0.08 [-0.02, 0.18] | 13 (54) | 0.20 [-0.04, 0.44] |
| Generic MBP | 6 (11) | 0.15 [-0.13, 0.43] | 5 (29) | *0.42* [0.12, 0.71] |

*Blue italics* = *df* < 4 (*p*-value not reliable). No estimate with satisfactory *df* reached statistical significance for any subgroup. **Abbreviations** – MBP = Mindfulness-based program; *K* = Number of studies; ES = Effect size; *g* = Hedge’s standardized mean difference (positive values imply improvement); CI = Confidence interval; *df* = Degrees of freedom. **Superscript** – *Includes an additional inactive arm from each of the five studies which featured both active and inactive comparators.

**Table S10**

Exploratory meta-analyses of MBP type broken down by clinical status

| **MBP type** | **Clinical** | | **Non-clinical** | |
| --- | --- | --- | --- | --- |
|  | **K (*N* ES)** | **ES (*g*) [95% CI]** | **K (*N* ES)** | **ES (*g*) [95% *CI*]** |
| MBSR/MBCT | 6 (18) | 0.04 [-0.54, 0.62] | 6 (21) | 0.05 [-0.33, 0.43] |
| Modified MBP | 11 (57) | **0.13** [0.04, 0.23] | 12 (45) | 0.16 [-0.08, 0.40] |
| Generic MBP* | - | - | 9 (37) | **0.31** [0.12, 0.51] |

Effects in **bold** reached statistical significance (*p* < .05). **Abbreviations** – MBP = Mindfulness-based program; *K* = Number of studies; ES = Effect size; *g* = Hedge’s standardized mean difference (positive values imply improvement); CI = Confidence Interval. **Superscript** – *There was only one Generic MBP study of a clinical sample, and thus data could not be meta-analyzed. **NB:** the main estimate for generic MBPs in the main paper might be ‘inflated’, given that it predominantly represents non-clinical samples, for which effect sizes were typically larger.

# Supplementary References

Baird, B., Mrazek, M. D., Phillips, D. T., & Schooler, J. W. (2014). Domain-Specific Enhancement of Metacognitive Ability Following Meditation Training. *143*(5), 1972-1979. <https://doi.org/10.1037/a0036882>

Becerra, R., Dandrade, C., & Harms, C. (2017, 2017/09/01). Can Specific Attentional Skills be Modified with Mindfulness Training for Novice Practitioners? *Current Psychology, 36*(3), 657-664. <https://doi.org/10.1007/s12144-016-9454-y>

Bowden, D., Gaudry, C., An, S. C., & Gruzelier, J. (2012, 2011/12/15). A Comparative Randomised Controlled Trial of the Effects of Brain Wave Vibration Training, Iyengar Yoga, and Mindfulness on Mood, Well-Being, and Salivary Cortisol. *Evidence-Based Complementary and Alternative Medicine, 2012*, 234713. <https://doi.org/10.1155/2012/234713>

Bubb, R. (2014). *Mindfulness-Based Stress Reduction as a Cognitive Intervention in Older Adults: A Feasibility Study* (Publication Number 3644754) [Ph.D., Bryn Mawr College]. ProQuest Dissertations & Theses Global. Ann Arbor. <https://search.proquest.com/docview/1625052533?accountid=14511>

Fam, J., Sun, Y., Qi, P., Lau, R. C., Feng, L., Kua, E. H., & Mahendran, R. (2020, Apr). Mindfulness practice alters brain connectivity in community-living elders with mild cognitive impairment. *Psychiatry Clin Neurosci, 74*(4), 257-262. <https://doi.org/10.1111/pcn.12972>

Ives-Deliperi, V. L., Howells, F., Stein, D. J., Meintjes, E. M., & Horn, N. (2013, Sep 25). The effects of mindfulness-based cognitive therapy in patients with bipolar disorder: a controlled functional MRI investigation. *J Affect Disord, 150*(3), 1152-1157. <https://doi.org/10.1016/j.jad.2013.05.074>

Jensen, C. G., Vangkilde, S., Frokjaer, V., & Hasselbalch, S. G. (2012). Mindfulness training affects attention--or is it attentional effort? *, General. 141*(1), 106-123.

Jermann, F., Van der Linden, M., Gex-Fabry, M., Guarin, A., Kosel, M., Bertschy, G., Aubry, J. M., & Bondolfi, G. (2013). Cognitive functioning in patients remitted from recurrent depression: Comparison with acutely depressed patients and controls and follow-up of a Mindfulness-Based Cognitive Therapy Trial. *Cognitive Therapy and Research, 37*(5), 1004-1014. <https://doi.org/10.1007/s10608-013-9544-1>

Johns, S. A., Von Ah, D., Brown, L. F., Beck-Coon, K., Talib, T. L., Alyea, J. M., Monahan, P. O., Tong, Y., Wilhelm, L., & Giesler, R. B. (2016, Jun). Randomized controlled pilot trial of mindfulness-based stress reduction for breast and colorectal cancer survivors: effects on cancer-related cognitive impairment. *J Cancer Surviv, 10*(3), 437-448. <https://doi.org/10.1007/s11764-015-0494-3>

Larouche, E., Hudon, C., & Goulet, S. (2018). Investigating a Mindfulness-Based Intervention as an Attentional Network Training to Improve Cognition in Older Adults with Amnestic Mild Cognitive Impairment: A Randomized-controlled Trial. *Neuropsychologie Clinique et Appliquée, 2*(Autome/Fall).

Lebares, C. C., Guvva, E. V., Olaru, M., Sugrue, L. P., Staffaroni, A. M., Delucchi, K. L., Kramer, J. H., Ascher, N. L., & Harris, H. W. (2019, May 3). Efficacy of Mindfulness-Based Cognitive Training in Surgery: Additional Analysis of the Mindful Surgeon Pilot Randomized Clinical Trial. *JAMA Netw Open, 2*(5), e194108. <https://doi.org/10.1001/jamanetworkopen.2019.4108>

MacCoon, D. G., MacLean, K. A., Davidson, R. J., Saron, C. D., & Lutz, A. (2014). No Sustained Attention Differences in a Longitudinal Randomized Trial Comparing Mindfulness Based Stress Reduction versus Active Control. *Plos One, 9*(6), e97551. <https://doi.org/10.1371/journal.pone.0097551>

Morris, S. B. (2007, 2008/04/01). Estimating Effect Sizes From Pretest-Posttest-Control Group Designs. *Organizational Research Methods, 11*(2), 364-386. <https://doi.org/10.1177/1094428106291059>

Moynihan, J. A., Chapman, B. P., Klorman, R., Krasner, M. S., Duberstein, P. R., Brown, K. W., & Talbot, N. L. (2013). Mindfulness-based stress reduction for older adults: effects on executive function, frontal alpha asymmetry and immune function. *Neuropsychobiology, 68*(1), 34-43. <https://doi.org/10.1159/000350949>

Tang, V., Poon, W. S., & Kwan, P. (2015, Sep 29). Mindfulness-based therapy for drug-resistant epilepsy: An assessor-blinded randomized trial. *Neurology, 85*(13), 1100-1107. <https://doi.org/10.1212/wnl.0000000000001967>

Webb, L., Perry-Parrish, C., Ellen, J., & Sibinga, E. (2018, Jun). Mindfulness instruction for HIV-infected youth: a randomized controlled trial. *AIDS Care, 30*(6), 688-695. <https://doi.org/10.1080/09540121.2017.1394434>

Wetherell, J. L., Hershey, T., Hickman, S., Tate, S. R., Dixon, D., Bower, E. S., & Lenze, E. J. (2017, Jul). Mindfulness-Based Stress Reduction for Older Adults With Stress Disorders and Neurocognitive Difficulties: A Randomized Controlled Trial. *J Clin Psychiatry, 78*(7), e734-e743. <https://doi.org/10.4088/JCP.16m10947>
